# Phylogenetic systematics of *Butyrivibrio* and *Pseudobutyrivibrio* genomes illustrate vast taxonomic diversity, open genomes and an abundance of carbohydrate-active enzyme family isoforms

**DOI:** 10.1099/mgen.0.000638

**Published:** 2021-10-04

**Authors:** Sara E. Pidcock, Timofey Skvortsov, Fernanda G. Santos, Stephen J. Courtney, Karen Sui-Ting, Christopher J. Creevey, Sharon A. Huws

**Affiliations:** ^1^​ School of Biological Sciences and Institute for Global Food Security, 19 Chlorine Gardens, Queen’s University Belfast, Belfast BT9 5DL, UK; ^2^​ School of Pharmacy, Medical Biology Centre, 97 Lisburn Road, Queen’s University Belfast, Belfast BT9 7BL, UK

**Keywords:** *Butyrivibrio*, evolution, pangenome, *Pseudobutyrivibrio*, rumen, taxonomy

## Abstract

*
Butyrivibrio* and *Pseudobutyrivibrio* dominate in anaerobic gastrointestinal microbiomes, particularly the rumen, where they play a key role in harvesting dietary energy. Within these genera, five rumen species have been classified (* Butyrivibrio fibrisolvens *, *Butyrivibrio hungatei *, *Butyrivibrio proteoclasticus *, *Pseudobutyrivibrio ruminis* and *Pseudobutyrivibrio xylanivorans *) and more recently an additional *Butyrivibrio* sp. group was added. Given the recent increase in available genomes, we re-investigated the phylogenetic systematics and evolution of *Butyrivibrio* and *Pseudobutyrivibrio *. Across 71 genomes, we show using 16S rDNA and 40 gene marker phylogenetic trees that the current six species designations (* P. ruminis *, *P. xylanivorans *, *B. fibrisolvens *, *Butyrivibrio* sp., *B. hungatei* and *B. proteclasticus*) are found. However, pangenome analysis showed vast genomic variation and a high abundance of accessory genes (91.50–99.34 %), compared with core genes (0.66–8.50 %), within these six taxonomic groups, suggesting incorrectly assigned taxonomy. Subsequent pangenome accessory genomes under varying core gene cut-offs (%) and average nucleotide identity (ANI) analysis suggest the existence of 42 species within 32 genera. Pangenome analysis of those that still group within *B. fibrisolvens *, *B. hungatei* and *P. ruminis *, based on revised ANI phylogeny, also showed possession of very open genomes, illustrating the diversity that exists even within these groups. All strains of both *Butyrivibrio* and *Pseudobutyrivibrio* also shared a broad range of clusters of orthologous genes (COGs) (870), indicating recent evolution from a common ancestor. We also demonstrate that the carbohydrate-active enzymes (CAZymes) predominantly belong to glycosyl hydrolase (GH)2, 3, 5, 13 and 43, with numerous within family isoforms apparent, likely facilitating metabolic plasticity and resilience under dietary perturbations. This study provides a major advancement in our functional and evolutionary understanding of these important anaerobic bacteria.

## Data Summary

*Pseudobutyrivibrio xylanivorans* MZ8 was genome sequenced in this study and the sequence submitted to GenBank (BioProject number PRJNA563299) (https://www.ncbi.nlm.nih.gov/bioproject/?term=PRJNA563299). Supplementary Material can be found with the online version of this article and on FigShare (https://doi.org/10.6084/m9.figshare.14785131.v1).

**Impact Statement**

Previous studies have suggested that immense intra- and inter-genetic variation lies within the genera *Butyrivibrio* and *Pseudobutyrivibrio *. Whether this is an artefact of inconsistent taxonomic approaches or a legitimate occurrence within the genera is not known. As such, their current taxonomic designations should be reviewed using current methodologies. This is also particularly timely as recently there has been a major increase in deposited available genomes for these genera. Consequently, this study reviews the taxonomy of *Butyrivibrio* and *Pseudobutyrivibrio* using computational approaches [including pangenomics, average nucleotide identity (ANI) and gene orthology] to determine the validity of their current taxonomy. We found that the current six species of *Butyrivibrio* and *Pseudobutyrivibrio* underrepresent the true taxonomic diversity and suggest based on ANI and coverage that the 71 genomes used in this study constitute 42 species within 32 genera, with those that still group within *Butyrivibrio fibrisolvens *, *Butyrivibrio hungatei* and *Pseudobutyrivibrio ruminis* based on revised ANI phylogeny showing very open genomes. As such, we suggest re-evaluating the species and genus designations of the strains included in this study. Despite genetic dissimilarity, all strains appear to maintain a similar broad functional profile within the rumen and share a broad range of clusters of orthologous genes (COGs), indicating fairly recent evolution from a common ancestor. Strains also possess an abundance of glycosyl hydrolase isoforms, which may afford them greater metabolic plasticity *in vivo*.

## Introduction

The definition of ‘species’ in bacteria or archaea is contentious, with some believing that the search for a single, natural way to divide bacteria into species is futile [1, 2]. Historically, the most important characteristics in terms of taxonomic markers were morphology, growth requirements and pathogenic potential. At the beginning of the 20th century, more biochemical and physiological markers were added to this list, followed by chemotaxonomy, numerical taxonomy and DNA–DNA hybridization in the mid-late 20th century. More recently, we have also used genotypic analyses, multilocus sequence analyses, average nucleotide identity (ANI), whole-genome analyses, etc. [3, 4]. 16S rDNA became a popular metric in the 1980s, with organisms sharing greater than 97 % 16S rDNA being classified as a single species [5]. This was further developed to whole-genome alignments [6] and phylogenetic clustering [7], both facing scrutiny for their seemingly arbitrary cut-off values [8]. 16S rDNA was also criticised on the basis that only a single gene is used as a point of comparison [9], prompting the development of sets of universal marker genes, which were proposed to form a more resolved phylogeny, but these constructions were far more computationally demanding [10]. It is inevitable that some degree of subjectivity is seen with respect to taxonomy, and consequently the same group of organisms can be sorted and arranged in many different ways [4, 11]. More recently, pangenomic analyses (those which look at shared core genes, accessory genes that confer variability, and the combination of these as the pangenome) have also been suggested as potential methods for defining bacterial species [12–14].

The rumen microbiome is taxonomically ambiguous, with horizontal gene transfer being rife due to the intense proximity that it provides [15]. Consequently, our understanding of both the taxonomy and function of the constituent microbes remains vague as it is in constant flux. Recently, our understanding has been enhanced through the Hungate collection [16], which comprise 501 rumen microbial genomes. Recent studies show that a core microbiome including *Prevotella *, *Butyrivibrio* and *Ruminococcus* can be found in ruminants globally [17]. Nonetheless, the taxonomy of *Butyrivibrio* and *Pseudobutyrivibrio* remains a topic of debate.

**Fig. 1.**
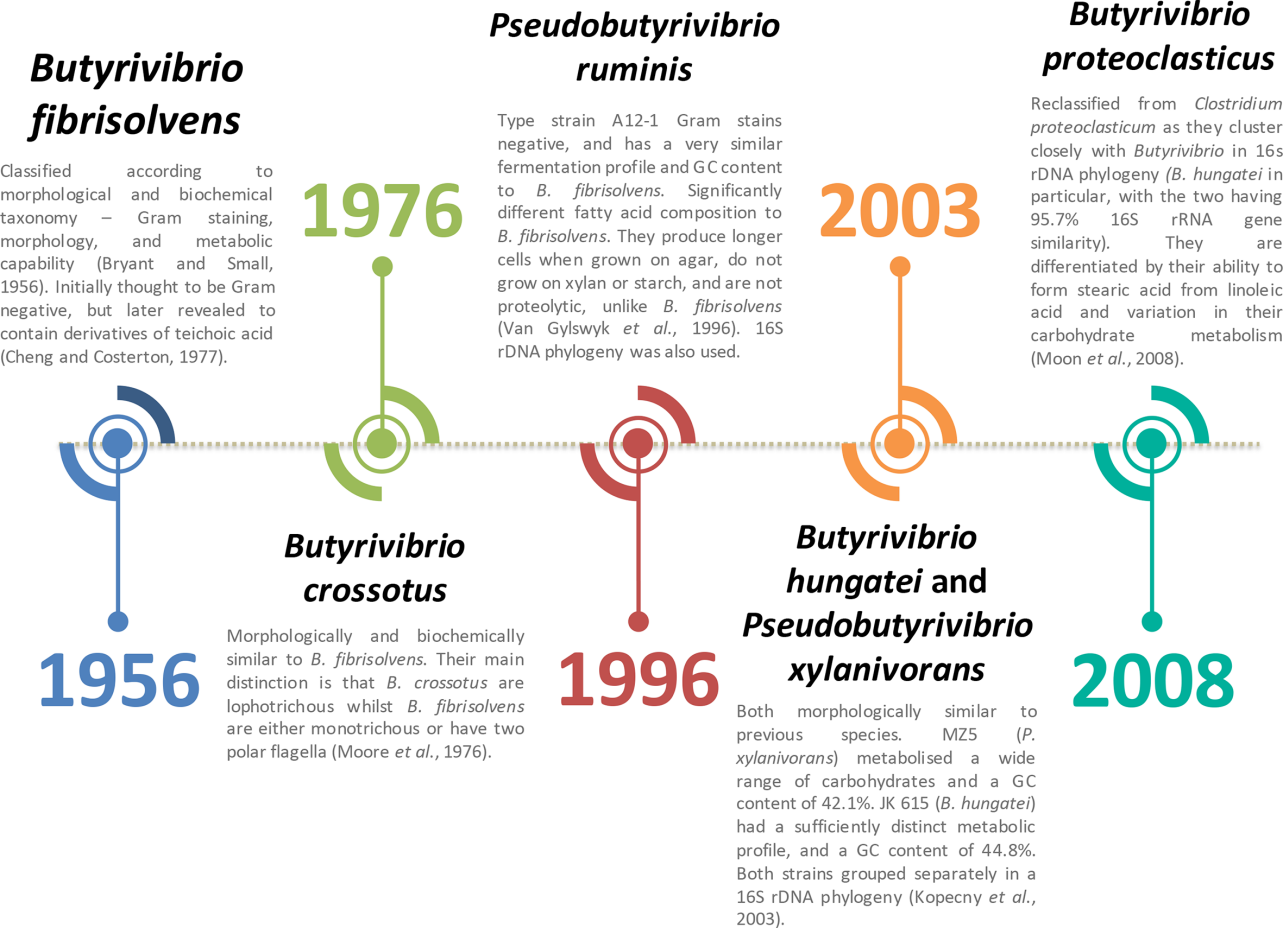
Chronological identification and classification of *Butyrivibrio* and *Pseudobutyrivibrio *.

*Butyrivibrio* were first described in 1956 using classical morphological and biochemical taxonomy; they are motile, Gram-positive, slightly curved rods that produce large amounts of butyric acid via glucose fermentation [18–20]. It was noted upon discovery that extensive variation within the genus may lead to difficulties in defining species–specific patterns [18, 19, 21]. They were called *Butyrivibrio fibrisolvens *, after their importance in the digestion of fibre in ruminant feed [18, 19] via carbohydrate-active enzymes (CAZymes) [22]. New strains were routinely classified as *B. fibrisolvens* despite morphological and genetic diversity [23] until 1976, when *Butyrivibrio crossotus *, a predominantly human isolate, was first described [24]. Thereafter, four *Butyrivibrio* species were defined: *B. fibrisolvens *, *B. crossotus *, *Butyrivibrio hungatei* and *Butyrivibrio proteoclasticus* (Fig. 1) [19, 23–25]. In 2008, *B. proteoclasticus* was reclassified, originally being *Clostridium proteoclasticum *, based on phylogenetic placement, DNA G+C content and physiological traits [25]. In 1996, a bacterium was isolated that resembled *B. fibrisolvens *, but varied sufficiently based on 16S rDNA, G+C content and cellular fatty acid content, and was named *Pseudobutyrivibrio ruminis* [26]. Later in 2003, *Pseudobutyrivibrio xylanivorans* was classified based upon fermentation characteristics, DNA G+C content and 16S rDNA dissimilarity to *Butyrivibrio* spp. [23]. Despite these additional species being named, *Butyrivibrio* and *Pseudobutyrivibrio* still possess untapped phylogenetic and genetic diversity with a sixth undefined *Butyrivibrio* sp. grouping identified recently [27].

The aims of this study were to re-investigate phylogeny, gene-level functional divergence and evolution in predominantly ruminal *Butyrivibrio* and *Pseudobutyrivibrio* using all publicly available genomes from pure cultures. This study also investigated gene-centric evolution in the ruminal *Butyrivibrio* and *Pseudobutyrivibrio* in relation to their pangenomes. In parallel, we also classified their carbohydrate degrading capacity. Many of the bacterial genomes in the recently expanded Hungate collection are from the genera *Butyrivibrio* and *Pseudobutyrivibrio *, which makes this study timely, enabling a paradigm shift in our fundamental understanding of these genera.

## Methods

### Genomes used in this study

Seventy-one genomes of *Butyrivibrio */* Pseudobutyrivibrio* isolates were obtained from the Hungate collection (Joint Genome Institute) [16] and including one additional strain, *P. xylanivorans* MZ8 (obtained from the Rowett Research Institute, University of Aberdeen, Aberdeen, UK), genome sequenced (Table S1, available with the online version of this article) by ourselves using MicrobesNG (https://microbesng.uk/) (sequenced on the Illumina HiSeq 2500 platform, using 2×250 bp paired-end reads and with 30× coverage) and submitted to GenBank (BioProject number PRJNA563299). The data were put through MicrobesNG’s standard analysis pipeline, which included strain identification by Kraken [28], *de novo* assembly of the reads by SPAdes [29] and Prokka V1.12 annotation [30]. However, we re-annotated all 71 genomes using Prokka v1.12 via the Galaxy platform [31] with a similarity *E* value cut-off of 1×10^−6^ to ensure all were annotated consistently.

### Phylogeny

16S rDNA sequences, obtained via Prokka annotations of the genomes, were aligned using the Aligner pipeline of the Ribosomal Database Project (RDP), release 11, update 5, September 30 2016 [32], and an approximately-maximum-likelihood phylogenetic tree with 1000 bootstrap repetitions was reconstructed using FastTree v2.1.10 [33]. An additional tree was reconstructed using 40 gene markers as per Wu and Eisen [34], and Creevey *et al*. [35]. Both trees were visualized by the Interactive Tree of Life (iTOL), changelog version 3.5.2 [36]. *Clostridium beijerinckii* NCIMB 8052 and *Lactobacillus acidophilus* NCFM were used as outgroups.

ANI was calculated using the PyANI script (available at https://github.com/widdowquinn/pyani/tree/version_0_2) [37]. Input sequences were in fasta format and were aligned using MUMmer (NUCmer). The comparisons were visualized by selecting for heatmap and dendrogram output.

### Pangenomics

Pangenomic analysis was carried out according to the classical six species taxonomy (* B. fibrisolvens *, *B. hungatei *, *B. proteoclasticus *, *Butyrivibrio* sp., *P. ruminis* and *P. xylanivorans *) to further define their phylogeny. Pangenomics was also used to define core and accessory genes within ANI-defined groupings containing at least four genome representatives. Core and accessory genomic fragments were identified from the Prokka annotated genomic sequences (.ffn files) using Spine v0.3.1 (http://vfsmspineagent.fsm.northwestern.edu/index_age.html) [38]. A range of other defined parameters (70–100 % similarity and present in 50–100 % of genomes) were evaluated, with 90 % [38–40], and the default value of 100 % core [41–43] definitions, using the default value of 85 % identity. Core values of 100 and 85% identity were subsequently used as the pangenomics parameters. Accessory elements were visualized using ClustAGE v0.8 and ClustAGE plot [38] for each group. The minimum accessory genomic element (AGE) size to represent in the ClustAGE plot was set to 1500 bp.

Core and accessory fasta files for individual strains were combined into the core genome and the accessory genome for their respective taxa based upon classical taxonomy and the ANI-refined taxonomy for the three groups with >4 genome representatives. We then prospected the *B. fibrisolvens* classical and ANI-defined core and accessory gene data for presence of the core orthologous genes used to conduct the 40 marker tree [11], using blastx and cut-off identity of 80 %. This was completed as these genes should be classified as core and as such were used as an estimate of the precision of core and accessory gene definitions for classical and ANI-refined taxonomy. Divergent bacteria will result in some core genes being incorrectly assigned as accessory. Core and accessory genes for the three ANI-refined groupings were uploaded to EggNOG [44] to determine genomic subsystem annotation. A stacked histogram was then made using these data to compare core and accessory functionality on a taxon level. Core and accessory gene G+C mol% content were also calculated from the Spine statistics files.

### Gene evolution

Putative gene orthology was determined using OrthAgogue [45] using the amino acid sequences of Prokka-annotated genes (.faa) from all 71 genomes. OrthAgogue was run with the parameters ‘-b -e 6’, which set the *E* value cut-off to 10^−6^ and forced OrthoMCL [46] emulation. We also ran OrthAgogue with an *E* value cut-off of 10^−5^ but the results were an *E* value cut-off to 10^−6^, which we decided to use for downstream analysis. All other OrthAgogue parameters were default. The clusters of orthologous genes (COGs) identified by OrthAgogue were turned into binary data, after which the lists of COGs were uploaded to UpSet [47] to visualize the intersections. It should be noted that output from OrthAgogue does not represent 1 : 1 gene orthology, rather it lists of genes that are part of clusters of orthologous genes.

### CAZymes

CAZymes were identified using the dbCAN metaserver [48] and annotated using the ‘mRNAs/CDSs/Metagenomes or short DNA seqs’ option, running hmmer with default setting of *E* value <1×10^−15^, coverage >0.35. Sequences were extracted using SAMtools v1.9 and each glycosyl hydrolase (GH) family was aligned using the ClustalOmega online server [49]. Trees were inferred by maximum likelihood using iq-tree v1.6.10, and visualized using iTOL. Homologue-based annotations were derived from dbCAN. Stacked histograms were produced for the most abundant GH families; in the case of the histogram with all 71 genomes displayed, and the histogram showing all six taxa, only GH families with over 100 total instances across all genomes were displayed. For the taxon-specific histograms, this number was 10. Pairwise per cent identity between GH sequences was determined by uploading amino acid sequences to the ClustalOmega online server, which produced a pairwise identity matrix.

### Metatranscriptome analysis

To check expression of the identified CAZyme isoforms in the rumen and *in vivo*, 20 publicly available metatranscriptomic datasets were taken from the National Center for Biotechnology Information Sequence Read Archive, under the accession number SRA075938. Datasets were composed of 150 bp paired-end reads from the Illumina HiSeq 2000 sequencer. fastq files were processed with multiqc [50] and reads were trimmed from 150 to 110 bp using trimmomatic software v0.36 [51]. Reads were aligned to the Hungate rumen genome dataset using bowtie2 v2.3.0 [52] using the settings ‘--very-sensitive-local’, allowing soft trimming, and a relaxed alignment; and ‘-k 497’. This produced SAM files, which were converted to BAM files using SAMtools v1.9 [53]. SAMtools v1.9 was used to filter all and the best alignment position for each read using the flag option ‘-F 260’. For each BAM file, FeatureCounts (from the subread package v2.0.0) [54] was used to calculate the number of reads that align within the boundaries of every predicted gene in the Hungate genomes. Read counts were converted into RPKM (reads per kilobase of transcript per million mapped reads) values. RPKM values of the CAZyme gene haplotypes were extracted from the entire expression count table. If a gene was found in a metatranscriptome (expressed to any degree) then it was visualized on the iTOL GH trees.

## Results

### Bacterial function

**Fig. 2.**
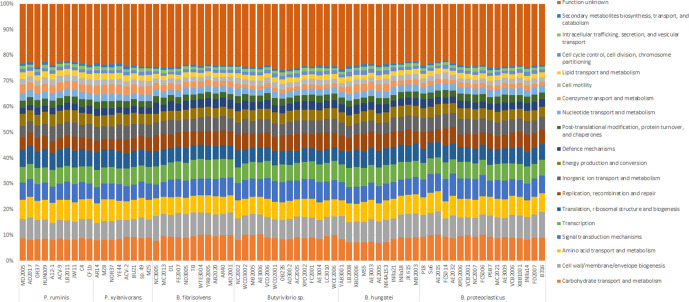
Functional annotation of the 71 *Butyrivibrio* and *Pseudobutyrivibrio* genomes used in this study. Gene functionality is sorted by colour, as indicated in the key. Annotation was performed using EggNOG [44] (http://eggnogdb.embl.de/#/app/emapper).

Functional analysis was completed for all 71 ruminal *Butyrivibrio* and *Pseudobutyrivibrio* genomes used in this study using EggNOG-mapper (Fig. 2, Table S1). Overall, strains have predominant functions relating to carbohydrate transport and metabolism (mean 8.91 %), and cell wall, membrane and envelope genesis (mean 8.05%). These stay constant across all strains (0.80 and 0.78 % sd). This general consistency can also be seen when the functional categories are compared at a genus level (Fig. S1).

### Phylogenetic analysis using 16S rDNA, 40 marker trees and ANI

**Fig. 3.**
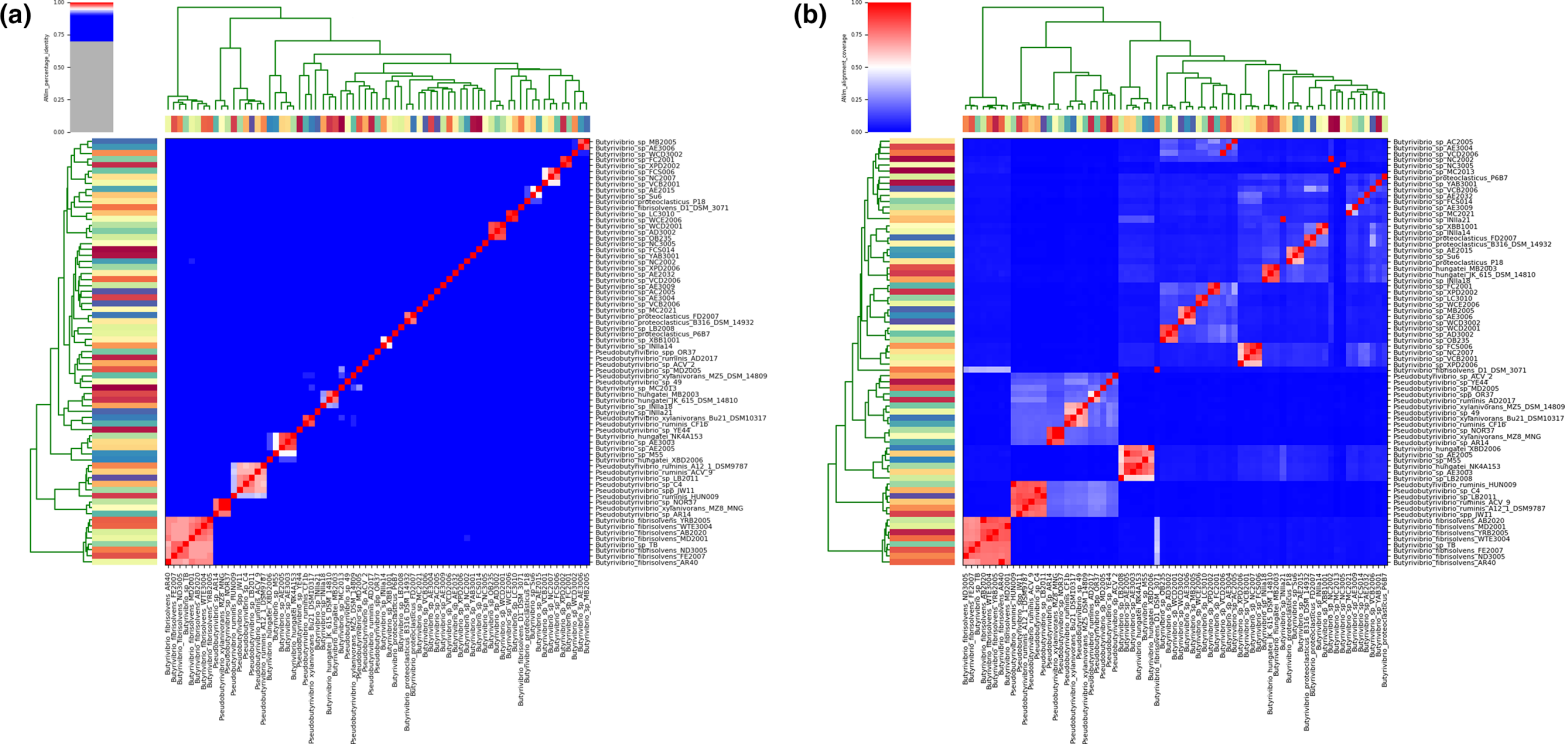
ANI comparison of 71 strains of *Butyrivibrio* and *Pseudobutyrivibrio* using pyani.py (https://github.com/widdowquinn/pyani/tree/version_0_2) with the MUMmer alignment option (**a**) (cells in the heat map that are coloured red have >95 % sequence similarity, whilst blue cells have <95 % similarity, and as nucleotide identity reaches 95 % the cells are coloured white). Alignment coverage of all strains using pyani.py with the MUMmer alignment option (**b**) (cells in the heat map that are coloured red have >50 % coverage, whilst blue cells have <50 % similarity, and as nucleotide identity reaches 95 % the cells are coloured white).

Phylogenetic trees based on 16S rDNA and 40 marker genes were reconstructed to examine phylogenetic relatedness (Figs S2 and S3, respectively). Both phylogenies form six groups that correspond to the classical taxonomy, including the additional group comprised entirely of strains allocated to *Butyrivibrio* but with no species designation (Table 1). It should be noted that *B. hungatei* forms a paraphyletic clade. Similar groupings can be seen again in the G+C-content-based scatter plot (Fig. S4), with the classical taxa *P. ruminis* and *P. xylanivorans* grouping very closely, as well as *B. hungatei* and *B. proteoclasticus *. However, ANI data showed that most strains have less than 95 % pairwise nucleotide identity (Fig. 3a), with small groups being above this threshold. Many of these strains have <50 % genome coverage, meaning that they align to each other for less than 50 % of their genome length (Fig. 3b). Indeed, ANI data suggests the existence of 32 genera and 42 species (Fig. 3a, b).

**Table 1.** Phylogeny of the 71 *Butyrivibrio* and *Pseudobutyrivibrio* strains used in this study, according to 16S rDNA, 40 marker tree and ANI analysis

Group	16S rDNA tree	40 marker tree	ANI
* B. fibrisolvens *	AB2020, AR40, D1, FE2007, MC2013, MC2021, MD2001, NC3005, ND3005, TB, WTE3004, YRB2005	AB2020, AR40, D1, FE2007, MC2013, MD2001, NC3005, ND3005, TB, WTE3004, YRB2005	AB2020, AR40, FE2007, MD2001, ND3005, TB, WTE3004, YRB2005
* B. hungatei *	AE2005, AE3003, INlla18, JK615, LB2008, M55, MB2003, NK4A153, VCB2006, XBD2006	AE2005, AE3003, INlla18, INlla21, JK615, LB2008, M55, MB2003, NK4A153, XBD2006, YAB3001	AE2005, AE3003, M55, NK4A153
* B. proteoclasticus *	AE2015, AE2032, AE3009, B316, FCS006, FCS014, FD2007, INlla14, INlla21, NC2007, P18, P6B7, Su6, VCB2001, XBB1001, XPD2006, YAB3001	AE2015, AE2032, AE3009, B316, FCS006, FCS014, FD2007, INlla14, MC2021, NC2007, P18, P6B7, Su6, VCB2001, VCB2006, XBB1001, XPD2006	
* Butyrivibrio* sp.	AC2005, AD3002, AE3004, AE3006, FC2001, LC3010, MB2005, OB235, VCD2006, WCD2001, WCD3002, WCE2006, XPD2002	AC2005, AD3002, AE3004, AE3006, FC2001, LC3010, MB2005, NC2002, OB235, VCD2006, WCD2001, WCD3002, WCE2006, XPD2002	
* P. ruminis *	A12-1, ACV-9, AD2017, C4, CF1b, HUN009, JW11, LB2011, NC2002, YE44	A12-1, ACV-9, AD2017, C4, CF1b, HUN009, JW11, LB2011, MD2005, OR37	A12-1, ACV-9, C4, HUN009, JW11, LB2011
* P. xylanivorans *	49, ACV-2, AR14, Bu21, MD2005, Mz5, Mz8, NOR37, OR37	49, ACV-2, AR14, Bu21, Mz5, Mz8, NOR37, YE44	

### Pangenomics

**Fig. 4.**
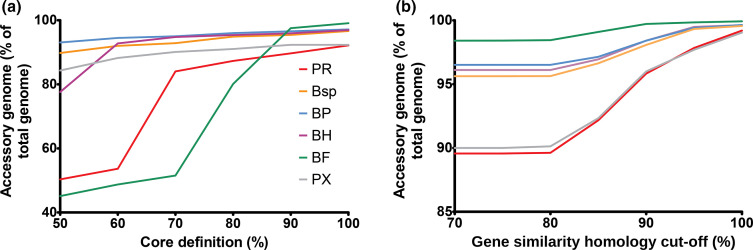
Effect of core definition percentage (**a**) and gene similarity homology (**b**) cut-off percentage on the pangenome composition of current *Butyrivibrio* and *Pseudobutyrivibrio* taxa as denoted by coloured lines: green being *B. fibrisolvens* (BF), purple being *B. hungatei* (BH), blue being *B. proteoclasticus* (BP), orange being *Butyrivibrio* sp. (Bsp), red being *P. ruminis* (PR) and grey being *P. xylanivorans* (PX).

Pangenomes were investigated using Spine software and a range of Spine cut-off parameters (core definition per cent and per cent nucleotide identity) were tested (Fig. 4a, b) . These data show that, as expected, the more stringent the core definition is (i.e. the more genomes of the total population that the gene must be in to be considered core), the more genes are considered to be accessory. These data also show the appearance of ‘bumps’ in the line graphs as parameters change, suggesting the presence of further taxonomical diversity within the classical clades (Fig. 4a, b). For downstream analysis, the default parameter of 100 % core definition was used as well as the default 85 % gene nucleotide identity cut-off based on choosing a mid-point that was both stringent but allowed some flexibility to account for the highly divergent nature of the bacteria (Table S1).

Using the classical taxa at a 100 % core definition, *B. fibrisolvens* has a mean core G+C content of 44.94 mol%, and an accessory G+C content of 40.57 mol%. *B. hungatei* has 44.73 and 41.04 mol% for mean core and accessory G+C contents, respectively; *B. proteoclasticus* 45.23 and 42.70 mol%, respectively; *Butyrivibrio* sp. 45.14 and 41.58 mol%, respectively; *P. ruminis* 43.39 and 39.06 mol%, respectively; and *P. xylanivorans* 43.69 and 39.12 mol%, respectively. *Pseudobutyrivibrio* strains had a greater difference than *Butyrivibrio* strains, with a mean difference of 4.32 mol% compared with 3.38 mol%. The greatest difference was seen in strains of *P. xylanivorans *, with a mean 4.57 mol% difference in G+C content. Core genes across each taxon appear to have a higher G+C mol% than their respective accessory genomes (with a mean of 44.57 mol% in the core genome, and 40.95 mol% in the accessory at a core definition of 90 %). This pattern can also be seen in the three main groups obtained using ANI (existing within *B. fibrisolvens *, *B. hungatei* and *P. ruminis* in the classical taxonomy), with the average G+C mol% for the three accessory pangenomes being 38.0 mol%, and the core being 41.55 mol% (Table S1). When based on the ANI groups (Table 1), core genomes were larger than that of classical taxa, comprising means of 59.62, 68.50, and 65.23 mol% for groups 1, 2 and 3 (* B. fibrisolvens *, *B. hungatei* and *P. ruminis *, respectively), although still illustrating very open genomes.

ClustAGE plots for classical taxa show high levels of genomic dissimilarity, with large numbers of AGEs being distributed throughout the genome (Figs S5–S10). The plot for *B. fibrisolvens* as a classical taxon shows gene fragments being absent in many genomes in places. This is particularly clear on occasion, for example, at the 750 kbp mark, with only 4 genomes out of 11 (AB2020, MC2013, NC3005 and D1) possessing an AGE here (Fig. S5). The ClustAGE plots for the PyANI groupings show sparser AGE fragments (due to a smaller accessory genome) but they appear to be similarly distributed (Figs S11–S13).

**Fig. 5.**
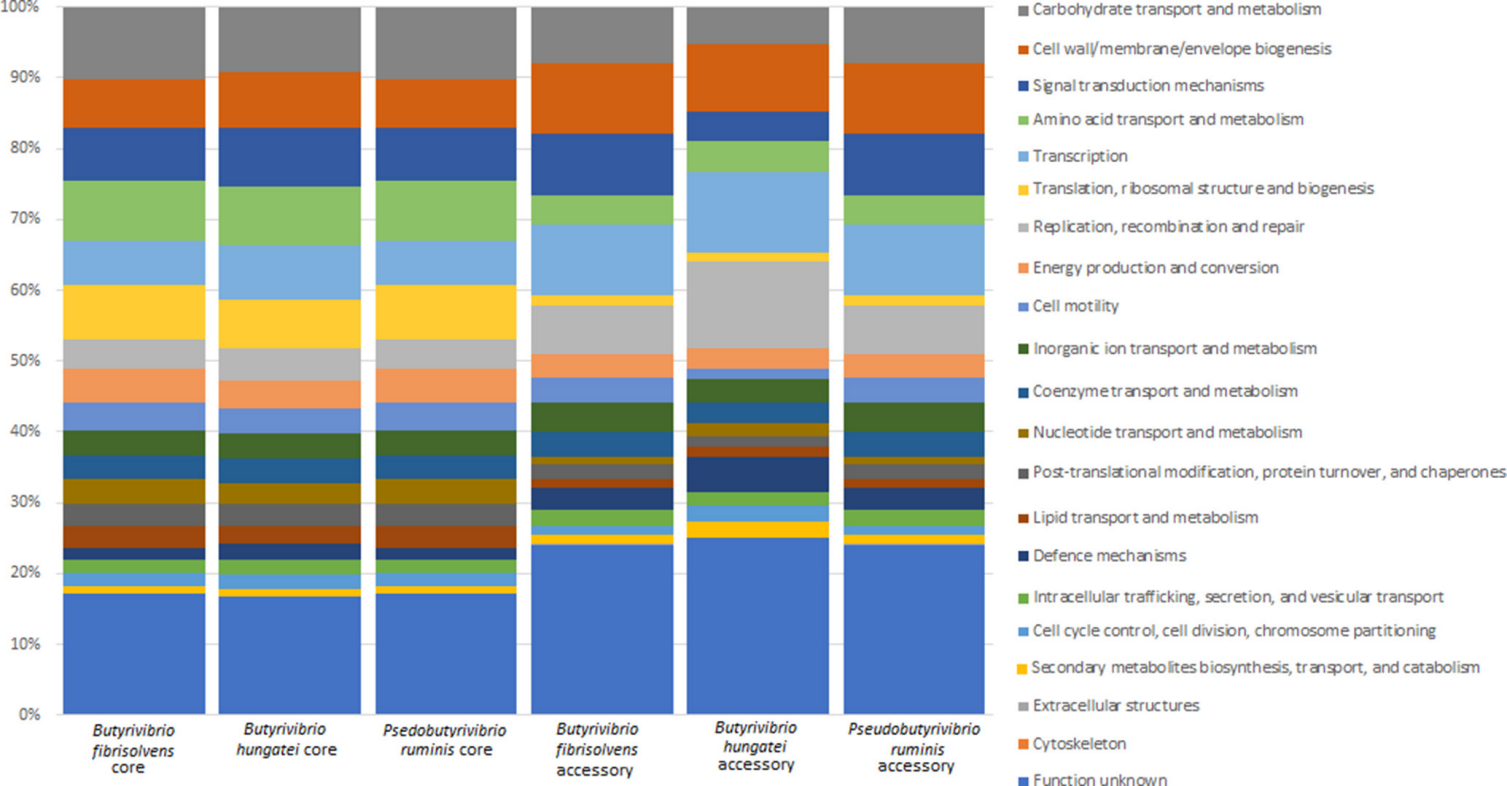
Functional annotations of the core and accessory genomes of the ANI-defined *B. fibrisolvens *, *B. hungatei* and *P. ruminis* groups.

Upon further checking of core and accessory gene assignments for the classical and ANI-defined *B. fibrisolvens* group for the 40 marker core orthologous genes used to reconstruct the 40 marker tree [11], it was noted that within the classically defined grouping that 11 of these were in the core and 22 in the accessory genes. Conversely, in the ANI-defined group, 26 were found within the core genes and none in the accessory genes. This again shows that the classical taxonomy is incorrect and provides further confidence in the fact that the members of the three ANI-defined groupings are correct, albeit they still possess open genomes. Functional annotation of the core and accessory genomes for the three groups also showed much similarity, with the only visual differences being that the proportion of unknown genes was higher in the accessory genome, and those related to translation, ribosomal structure and biogenesis were less numerous in the accessory genome (Fig. 5).

### Gene orthology and paralogy

**Fig. 6.**
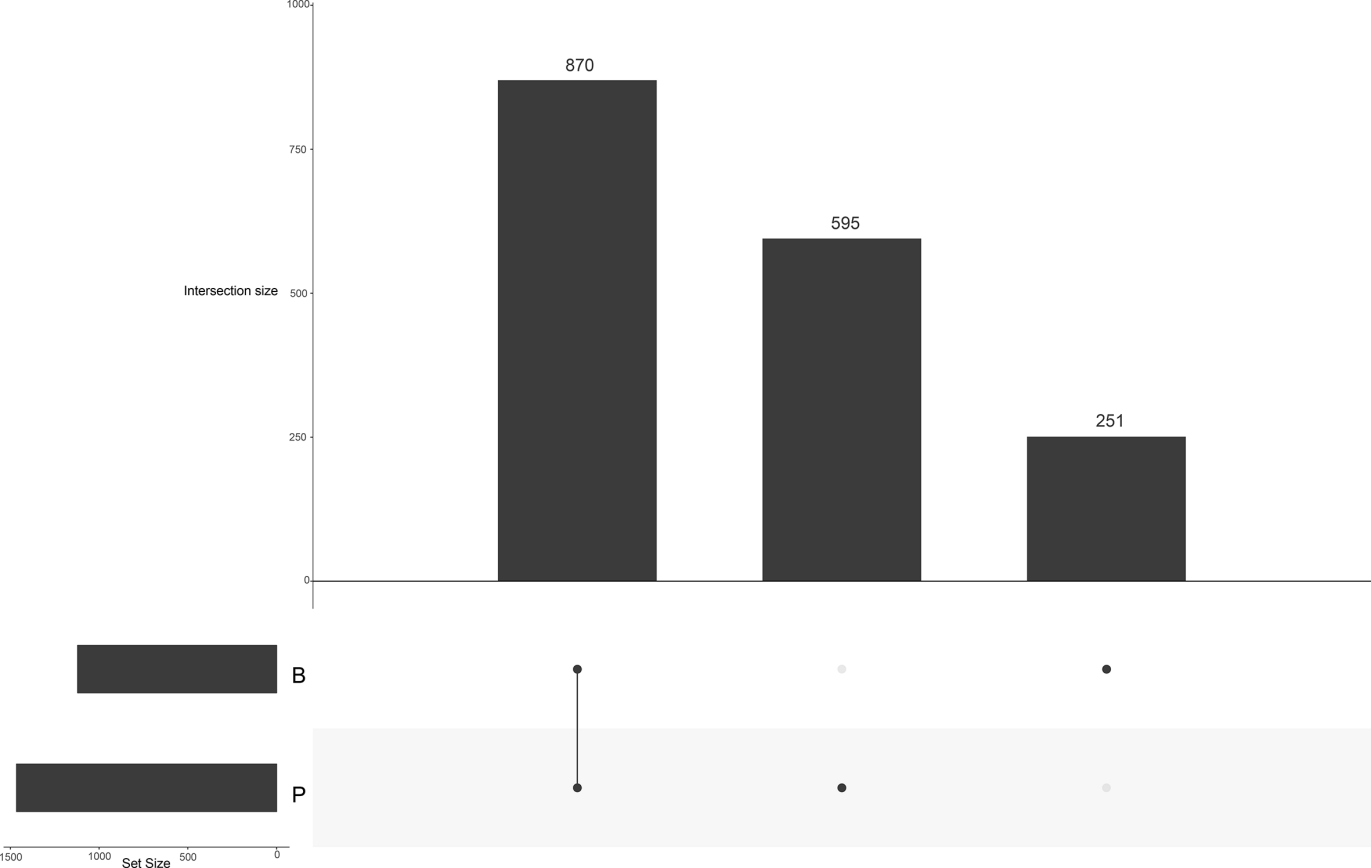
UpSet plot showing orthologous gene cluster intersections across the two genera, *Butyrivibrio* (B) and *Pseudobutyrivibrio* (P). Intersections are denoted by the corresponding bar.

In order to evaluate gene ancestry and evolution, OrthAgogue was used to identify orthologous gene affiliations. Both genera share the majority of their orthologous genes, with 870 COGs being common to the two (Fig. 6). As a genus, *Pseudobutyrivibrio* has more common orthologous genes than *Butyrivibrio *, with 343 and 223 genes, respectively. *Pseudobutyrivibrio* has more unique COGs (595) than *Butyrivibrio* (251) (Fig. 6). The genus *Butyrivibrio* had the most inparalogous clusters (2596), and *Pseudobutyrivibrio* the fewest (259) (Table S2). Further analysis based on core/accessory designations on the correctly defined ANI taxonomy showed that most core genes were orthologues: 55, 60 and 26 % for the *B. fibrisolvens *, *B. hungatei* and *P. ruminis* groups, respectively. Conversely, accessory orthologous genes constituted 36, 30 and 18 % of the total genes for the *B. fibrisolvens *, *B. hungatei* and *P. ruminis* groups, respectively. Core genes contained 1, 1 and 12% inparalogues for the *B. fibrisolvens *, *B. hungatei* and *P. ruminis* groups, respectively. Conversely, accessory inparalogues constituted 1, 2 and 1 % of the total genes for the *B. fibrisolvens *, *B. hungatei* and *P. ruminis* groups, respectively. Core genes also contained 3, 4 and 26 % co-orthologues for the *B. fibrisolvens *, *B. hungatei* and *P. ruminis* groups, respectively. Conversely accessory co-orthologues constituted 4, 3 and 17 % of the total genes for the *B. fibrisolvens *, *B. hungatei* and *P. ruminis* groups, respectively.

### GH haplotypes and evolution

**Fig. 7.**
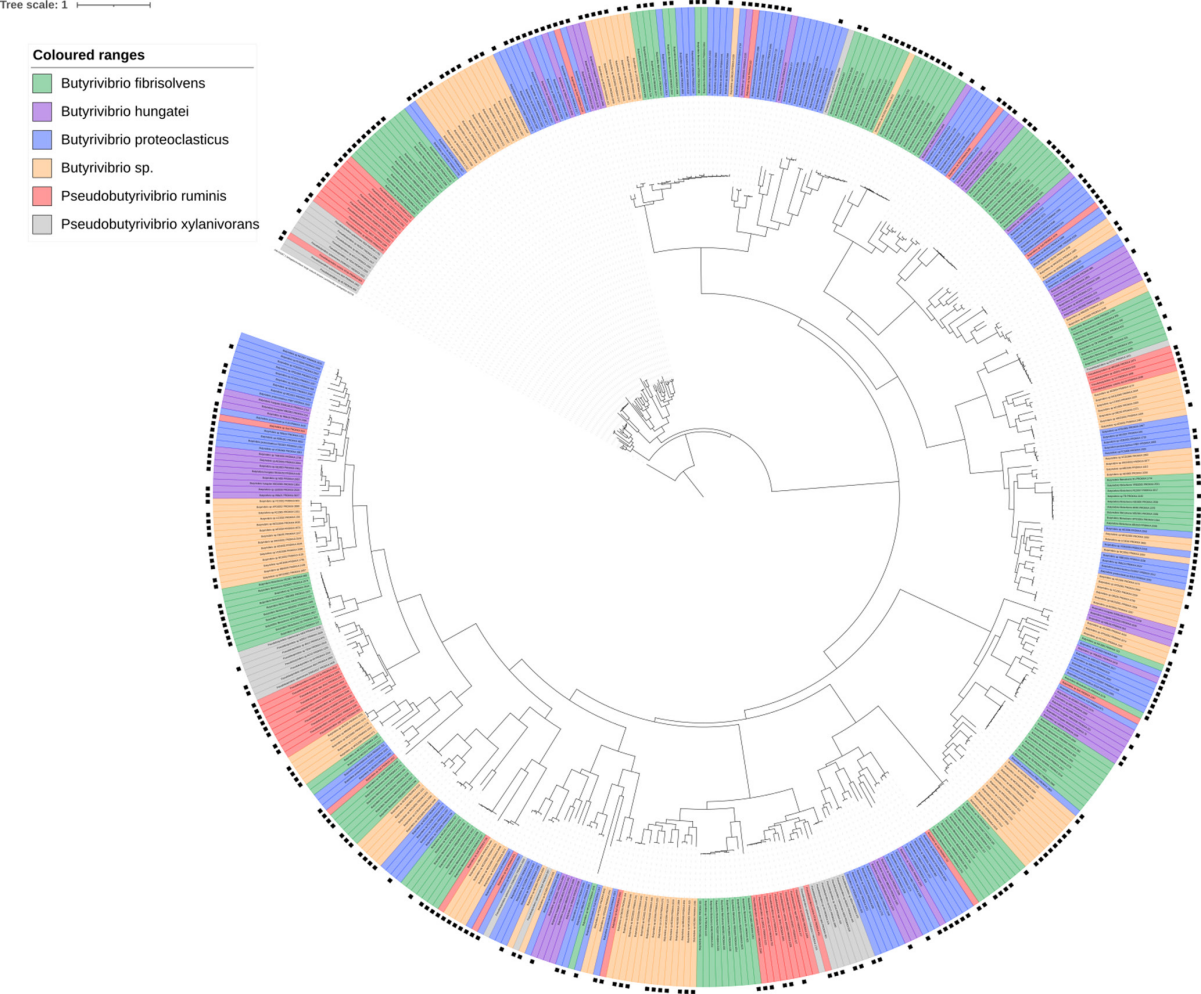
Phylogenetic tree showing the relatedness of all GH family 2 genes found in all 71 strains used in this analysis. presence of a black square on the outermost layer indicates that the gene was found to be present in the Shi *et al*. metatranscriptome dataset [55]. The tree is rooted using a β-galactosidase large subunit sequence from *L. acidophilus* NCFM, which is coloured in black. Tree scale indicates number of substitutions per site.

**Fig. 8.**
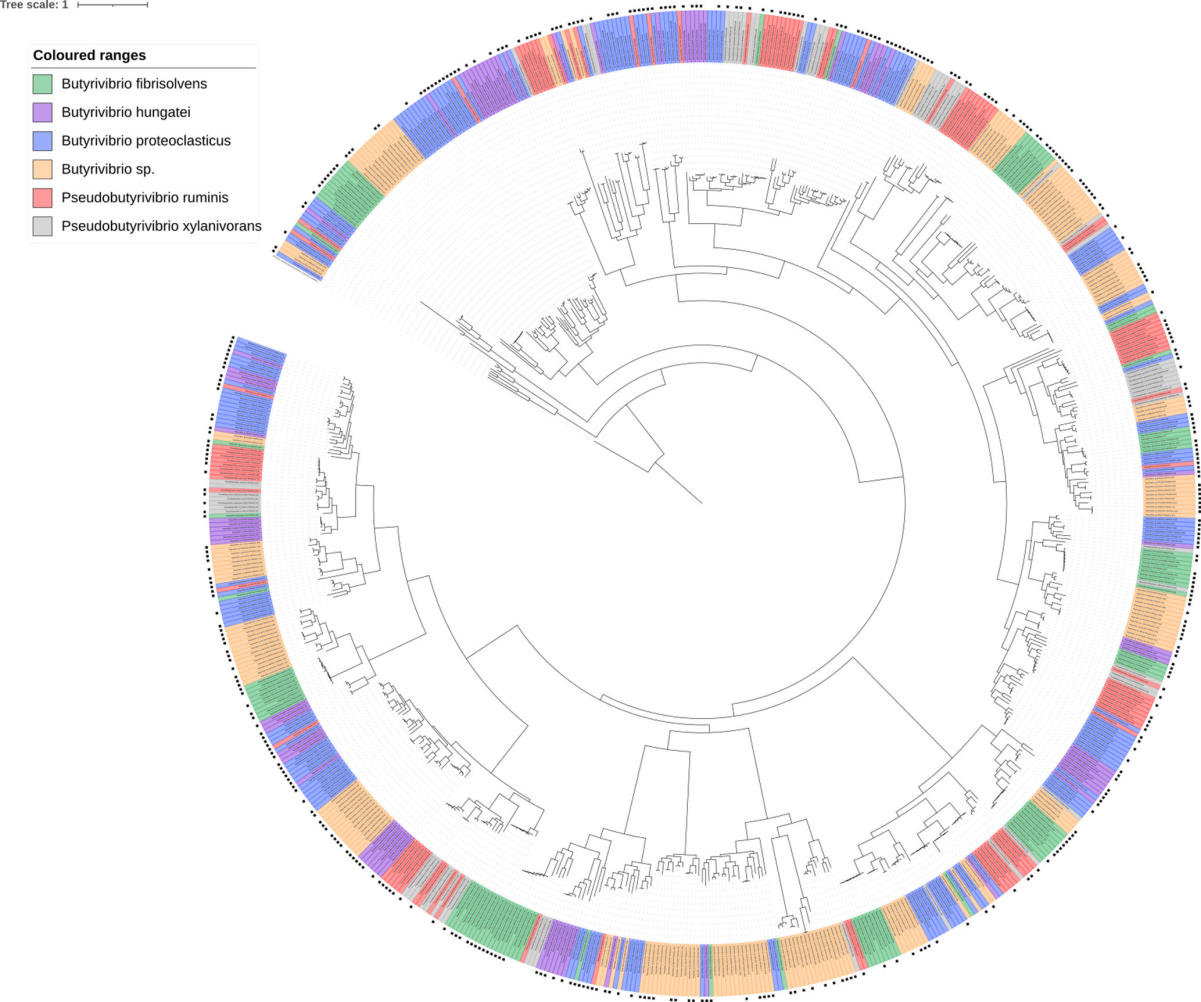
Phylogenetic tree showing the relatedness of all GH family 3 genes found in all 71 strains used in this analysis. The presence of a black square on the outermost layer indicates that the gene was found to be present in the Shi *et al*. metatranscriptome dataset [55]. The tree is rooted using a β-*N*-acetylhexosaminidase sequence from *L. acidophilus* NCTC13720, which is coloured in black.

**Fig. 9.**
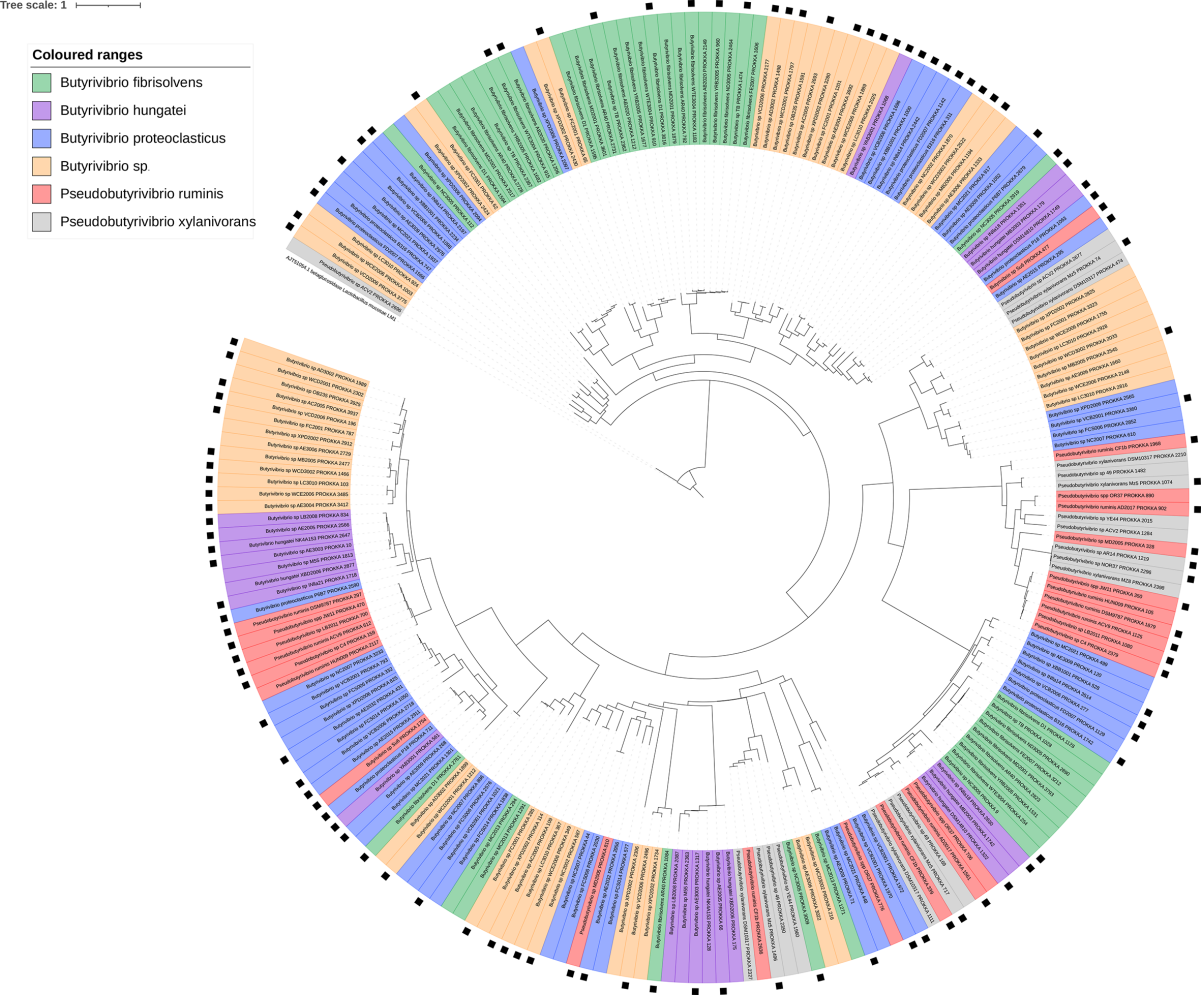
Phylogenetic tree showing the relatedness of all GH family 5 genes found in all 71 strains used in this analysis. The presence of a black square on the outermost layer indicates that the gene was found to be present in the Shi *et al*. metatranscriptome dataset [55]. The tree is rooted using a β-glucosidase sequence from *Lactobacillus mucosae* LM1, which is coloured in black.

**Fig. 10.**
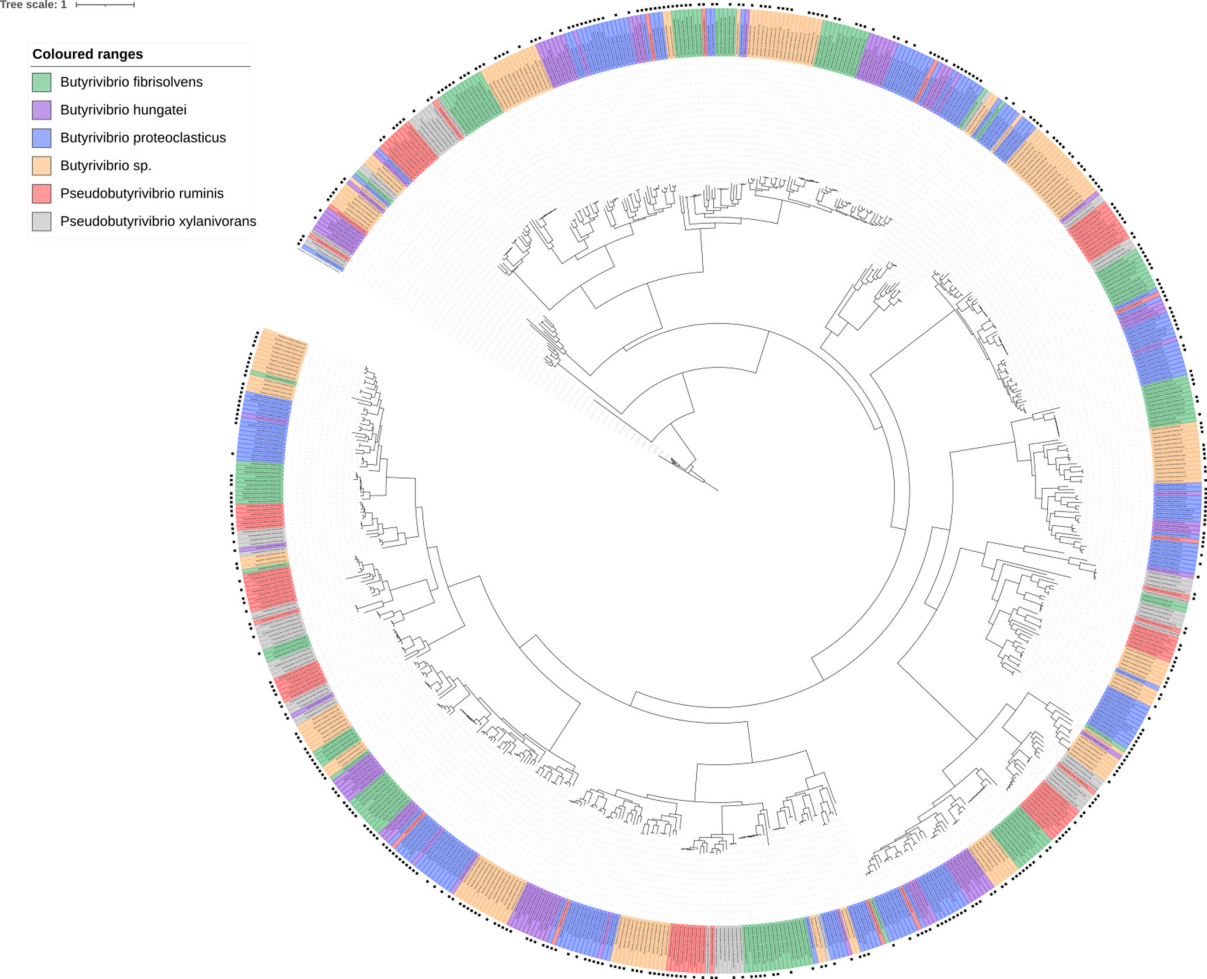
Phylogenetic tree showing the relatedness of all GH family 13 genes found in all 71 strains used in this analysis. The presence of a black square on the outermost layer indicates that the gene was found to be present in the Shi *et al*. metatranscriptome dataset [55]. The tree is rooted using a sucrose phosphorylase sequence from *L. acidophilus* NCFM, which is coloured in black.

**Fig. 11.**
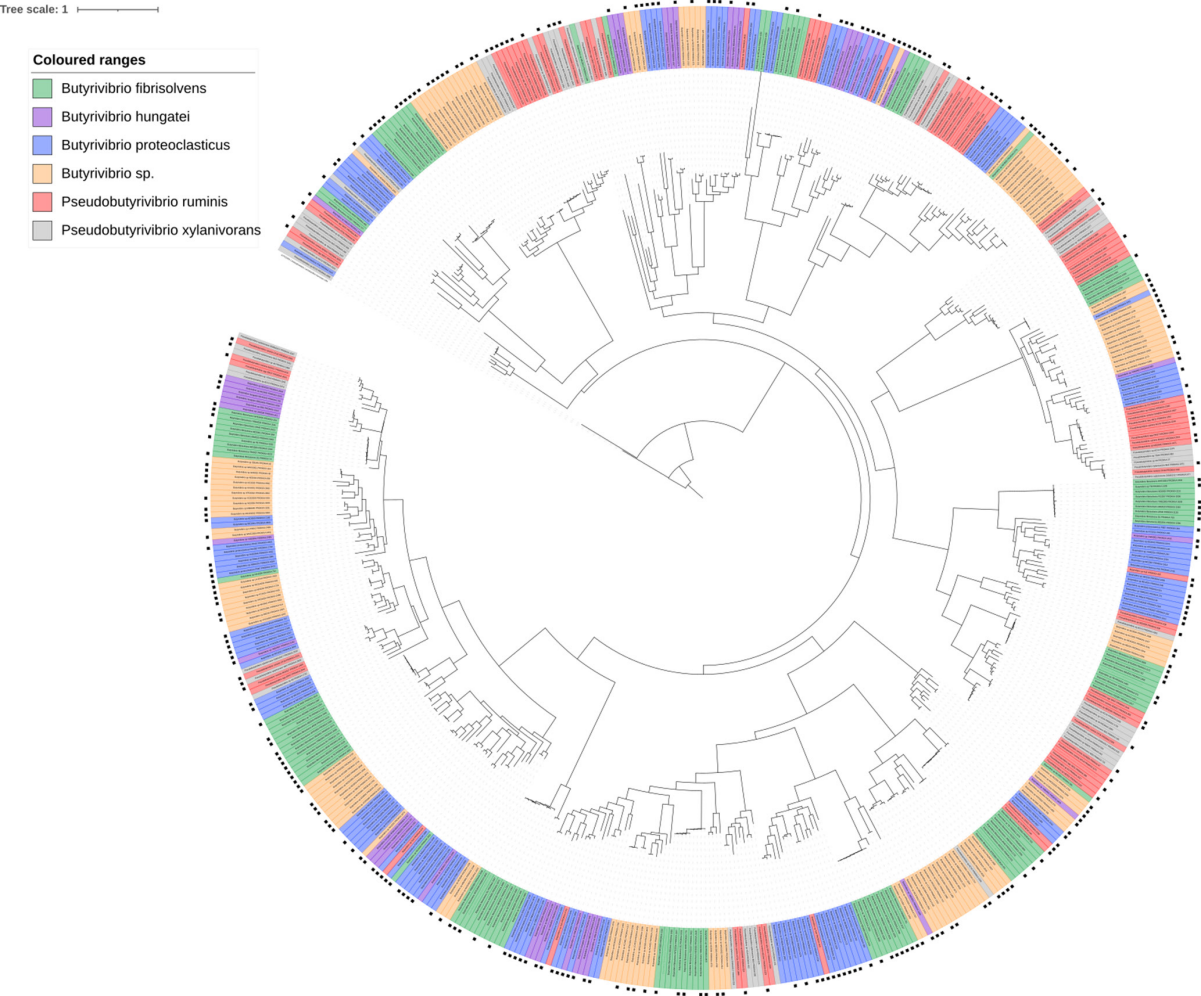
Phylogenetic tree showing the relatedness of all GH family 43 genes found in all 71 strains used in this analysis. The presence of a black square on the outermost layer indicates that the gene was found to be present in the Shi *et al*. metatranscriptome dataset [55]. The tree is rooted using a β-xylosidase sequence from *Lactobacillus mucosae* LM1, which is coloured in black.

Functional annotation of the GH families possessed by each strain showed a lot of similarity based on GH families and their abundances (Fig. S14). GH family phylogenetic trees show limited gene grouping, suggesting possession of a high degree of within-GH family enzyme isoform sequence diversity (Figs. 7–11). GH3 was the most abundant with 690 genes present, followed by GH13 with 681, GH43 with 543, GH2 with 463 and GH5 with 216 (Figs. 7–11, Supplementary Excel data 1). Pairwise comparisons of genes within these families showed extensive genomic diversity, with only 5.36 % of GH2 genes sharing a pairwise percentage identity of greater than 50 %. For GH3, this was 4.98 %, GH5 6.32 %, GH13 5.97 % and GH43 3.44 % (Supplementary Excel data 2). Irrespective of relatedness, for each GH family, the majority of genes within were found in at least 1 of the 20 metatranscriptomes within the Shi *et al*. dataset [55] (Figs 7–11, Supplementary Excel data 3). For GH2, 67.17 % of genes were found to be expressed, for GH3 62.87 %, GH5 56.28 %, GH13 66.57 % and GH43 59.41 %. Of these, many had an RPKM >1, illustrating that the GH isoforms discovered are not an anomaly of the assembly and are actively expressed.

## Discussion

The role that *Butyrivibrio* and *Pseudobutyrivibrio* play in the rumen is not yet fully understood; however, they are known to be heavily involved in the metabolism of carbohydrates [23, 56], proteins [57] and lipids [58]. Indeed, they dedicate a large proportion of their genetic capacity to the breakdown and reassembly of complex polysaccharides, with the resulting simple sugars undergoing fermentation to produce butyrate, a major source of energy for the ruminant [16, 22, 56, 59–61]. In this study, we show that *Butyrivibrio* and *Pseudobutyrivibrio* are more genetically diverse than their classical taxonomy suggests. They also possess open pangenomes, as shown by low core gene proportions, and numerous gene haplotypes within multiple CAZyme families, which we hypothesize may provide metabolic plasticity during dietary fluctuations. This study delves into the fundamental taxonomy, ecology and evolution of the *Butyrivibrio* and *Pseudobutyrivibrio* at a level not possible before the recent increase in available genomes [16].

A phylogenetic tree based on 40 conserved gene markers revealed groups that approximate to classical species, with the exception of *P. ruminis* strain CF1b. 16S rDNA phylogeny on fewer strains, performed by Kasperowicz *et al*. [62], showed that the CF1b strain groups closely with the type strain *P. ruminis* A12-1, which is concurrent with our own 16S rDNA findings. Whilst 16S rDNA analysis has historically been thought to be a reliable means of establishing distant relationships between organisms [63], extensive diversity has been found within the 16S rDNA of certain genomes [64, 65]. Furthermore, 16S rDNA has more recently been highlighted as providing poor resolution at a species level [9]. The 40 marker genes used are universal, single-copy genes that are highly conserved and appear to maintain a constant rate of horizontal transfer; as such, using these 40 markers is thought to provide a more resolved comparison [35]. Although both of these phylogenies broadly form the six classical taxa, some of these clades are not monophyletic, and as such this should not form the sole basis of their taxonomy. The ANI plot shows very little similarity overall between strains, yet several small groups with >95 % ANI can be observed; the first of these is comprised of eight strains that were allocated to *B. fibrisolvens* in the previous phylogenies, the second being six strains of *P. ruminis* and the third four strains of *B. hungatei *. The alignment coverage plot similarly shows extensive dissimilarity, with few groups having coverage of >50 %. Given that a 95 % ANI cut-off value is commonly used to delineate species, and 50 % coverage for genus [37], this suggests the presence of potentially 42 species in 32 genera. Wittouck *et al*. [66] used core nucleotide identity to reclassify the similarly structured *Lactobacillus* genus complex, stating that current bacterial taxonomy is not always consistent with bacterial evolutionary history, with some official taxa not being monophyletic (similar to the *Butyrivibrio* group). They proposed reclassification of 2459 genomes into 239 similar *de novo* species based on a 94 % core nucleotide identity cut-off. Following this, another study re-evaluated the taxonomic structure of *Lactobacillus *, stating that micro-organisms that are genetically distinct, as well as metabolically, ecologically and functionally diverse, were being grouped within the same genus, leading to high levels of genetic diversity. Using a combination of pangenomics, average amino acid identity (AAI), it was proposed that *Lactobacillus* be split into a further 24 genera, with the original emended genus bringing the total to 25 [67]. Given that ANI has been widely used for the delineation of species [9, 68, 69], we suggest that the genera *Butyrivibrio* and *Pseudobutyrivibrio* (and the species they currently encompass) be similarly split based on ANI into 42 species within 32 genera.

The increasing number of available bacterial genomes has allowed further research into microbial population genomics [70], which has revealed extensive intraspecific variability in prokaryotic genome content, and led to the study of pangenomes (all the gene families that have been found in the species as a whole), core genome (‘essential’ genes), and accessory genome (‘dispensable’ genes) [71]. This, alongside the analysis of orthologous genes (those derived from speciation events) and paralogous genes (those derived from duplication events), can give insight into the taxonomy and evolutionary divergence of a population. Pangenome analysis of our strains showed that, when analysed based on their classical taxon, *B. fibrisolvens* possesses the lowest percentage of core genes (2.45 %), and *P. ruminis* the highest (10.38 %), with both values being comparatively low, illustrating the low numbers of shared genes between strains. The previously acknowledged heterogeneity in *Butyrivibrio* [23, 24, 72] as it currently exists increases the probability of newly introduced genes being designated accessory. This leads to the artificial inflation of the accessory genome and the pangenome as a whole, if strains that are too dissimilar are allocated the same species, as may have often been the case given the historic tendency to classify new butyrate-producing bacteria as *B. fibrisolvens *, despite diversity and genetic relatedness [23, 73]. When based on the ANI re-classified groups, core genomes were larger, comprising means of 59.62, 68.50 and 65.23 % for groups 1, 2 and 3, within *B. fibrisolvens *, *B. hungatei* and *P. ruminis *, respectively. Further accuracy checking of accessory and core designations was conducted by prospecting genes in both for the 40 biomarker genes used to define the 40 marker phylogenetic tree [11]. These biomarker genes should be core in all strains and be orthologous in nature; therefore, their presence in the accessory genes suggests over-inflation of the accessory genome due to the presence of too much divergence in the strains. For the classical taxonomy, we found 22 of these genes within the classical taxonomy accessory genome of *B. fibrisolvens *, but none were found in the ANI-refined *B. fibrisolvens* group accessory genome. This suggests again that the classical taxonomy is incorrect and that the ANI re-definitions are highly likely to be more correct, although it should be noted that a reasonable diversity is still present in the ANI-defined groups. Indeed, these would still be considered to be open pangenomes when compared with, for example, *Pseudomonas aeruginosa *, whose core genes comprise 82–93 % of their genomes using a 90 % core definition [38]. Similarly, Lapierre and Gogarten [74] estimate that the typical bacterial genome would be composed of only 28 % accessory genes.

When based on ANI re-defined taxonomy, functions relating to translation, ribosomal structure and biogenesis were found mainly in the core genome. This is likely due to the fact that a core genome is thought to comprise essential gene families, i.e. housekeeping genes [71], which are more likely to be present in a wider range of less-related bacteria. Likewise, it is perhaps unsurprising that the accessory group has more unknown genes, as these are likely to be very unique as they have yet to be characterized.

G+C mol% is consistently higher in the core genomes of each of the 71 strains when the pangenome is based on classical taxa, and the 17 strains in three groups formed by ANI. It has been suggested that G+C-rich genome segments can occur as a result of biased gene conversion following recombination, whereby DNA repair of mismatched bases holds a bias towards G+C nucleotides [75]. If this is assumed to be correct, this G+C bias in core genes could be explained by their retention over accessory genes, which are more readily lost and exchanged [76]; the longer these core genes are retained, the more they will be subjected to DNA repair, resulting in an increasing amount of G+C bases being incorporated into the core genome. Indeed, a recent study that analysed 731 prokaryotic strains from 36 species, 28 genera and 10 phyla found that the G+C mol% of the core genome was significantly higher than that of the whole genome, and that the G+C mol% of the accessory genome was significantly lower than that of the whole genome [77].

In terms of their evolution, the majority of COGs are shared across both the genera *Butyrivibrio* and *Pseudobutyrivibrio *, with 870 clusters being common to all of them. The genus *Pseudobutyrivibrio* group share a high proportion of COGs (343) and the *Butyrivibrio* group share slightly fewer at 223. When the number of genes found as part of a cluster of orthologues, inparalogues and co-orthologues are separated into core and accessory for the ANI-defined groups, the core genomes appear to be largely composed of orthologues as would be expected for related strains. It should be noted, however, that many publications exist which show that accessory genomes contain the most orthologues, which is likely due to the fact that their strains are so divergent that the partitioning of core and accessory genes is more difficult, as was seen for the analysis completed for the classical *B. fibrisolvens* grouping.

GHs are involved in the breakdown of carbohydrates, including many plant polymers, and are broken down into 111 families (http://www.cazy.org/) on the basis of amino acid similarity [78]. The rumen microbiome is exposed to strong diet-driven selection pressures, meaning that they must constantly compete for available sources of nutrition during dietary fluctuations [79]. The topology of the GH family trees is indicative of multi-gene families, i.e. groups of genes that have arisen from a common ancestor by duplication, and indicates the presence of a multitude of different isoforms. It is not uncommon for extensive gene sequence variation to be found within a bacterial family, with the resulting enzymes having different substrate specificities and yielding different products [80]. Ohta [81] further states that many multi-gene families are present in large numbers within a genome due to an increased demand for their gene product, with genes either retaining their function or diverging. Pairwise comparisons of genes representing the same GH family also show the vast diversity in the gene sequences and possibly suggest that there is too much diversity to draw phylogenetic trees. Irrespective, both approaches illustrate the fact that diversity in high in the families. Bacteria in functionally demanding environments are thought to possess a vast array of functional isomers, allowing resilience under dietary perturbations [82]. The fact that such a large proportion of the GHs were found within the Shi *et al*. dataset [55] confirms that they are actively expressed within the rumen and not artefacts of the genome assembly.

### Conclusion

In conclusion, this study provides the most in-depth dataset on the phylogenetic systematics and evolution of the ruminal *Butyrivibrio* and *Pseudobutyrivibrio* to date. This study demonstrates remarkable genomic dissimilarity between strains that have previously been classified as the same species; this can be seen in the existence of outlier strains within the existing taxonomy in terms of phylogeny, G+C content, genome size and ANI, suggesting that they may be incorrectly classified. The genomes studied display very low per cent core genomes and high per cent accessory genomes when analysed in their current taxonomical groups. As such, we propose that their taxonomy be re-evaluated on the basis of their ANI to 42 species within 32 genera. Despite genomic variation, classical taxa appear to retain broadly similar high-level functional profiles, but possess a number of GH isoforms that we hypothesize facilitate metabolic plasticity and resilience under dietary perturbations.

## Supplementary Data

Supplementary material 1

Supplementary material 2

Supplementary material 3

Supplementary material 4
