# Supplementary material for: Phylogenetic systematics of Butyrivibrio and Pseudobutyrivibrio genomes illustrate vast taxonomic diversity, open genomes and an abundance of carbohydrate-active enzyme family isoforms
Source: Microb Genom. 2021 Oct 4;7(10):000638. doi: 10.1099/mgen.0.000638 (PMC8627218; doi:10.1099/mgen.0.000638)
Supplement: Supplementary material 1 [file mgen-7-0638-s001.pdf]

Supplementary data

Supplementary Table 1 - GC content (%) of the core and accessory genomes of 71 strains of *Butyrivibrio* and *Pseudobutyrivibrio* at 90% and 100% core definition (when analysed as their classical taxa, and when analysed as smaller groups sharing greater than 95% nucleotide identity), as determined by Spine (Ozer *et al*., 2014).

Phylogenetic clade	Strain ID	Core GC% (90%)	Acc GC % (90%)	Core GC% (100%)	Acc GC% (100%)	ANI group core GC%	ANI group accessory GC%
B. fibrisolvens	AB2020	44.82	40.69	44.85	40.73	41.74	38.92
	AR40	44.76	40.69	44.59	40.73	41.73	39.25
	D1	44.82	40.50	44.73	40.54	-	-
	FE2007	44.74	40.48	44.86	40.53	41.65	37.96
	MC2013	47.19	44.79	47.04	44.81	-	-
	MD2001	44.87	40.58	44.89	40.63	41.74	38.72
	NC3005	43.69	36.01	44.02	36.04	-	-
	ND3005	44.97	40.32	44.94	40.37	41.66	37.60
	TB	44.78	40.43	44.77	40.49	41.66	37.95
	WTE3004	44.75	40.72	44.95	40.76	41.73	38.98
	YRB2005	44.73	40.65	44.70	40.69	41.71	38.82
B. hungatei	AE2005	44.97	41.20	45.05	41.22	42.57	38.25
	AE3003	44.89	41.55	45.03	41.58	42.56	38.77
	INlla18	44.30	40.56	44.33	40.57	-	-
	INlla21	46.03	42.68	46.06	42.71	-	-
	JK615	44.23	40.30	44.14	40.31	-	-
	LB2008	44.71	41.15	44.83	41.19	-	-
	M55	44.94	41.29	45.03	41.33	42.61	38.59
	MB2003	44.17	40.28	44.08	40.29	-	-
	NK4A153	44.95	41.60	45.07	41.63	42.57	38.73
	XBD2006	44.76	41.37	44.92	41.41	-	-
	YAB3001	43.46	39.27	43.47	39.27	-	-
B. proteoclasticus	AE2015	43.84	40.33	43.73	40.35	-	-
	AE2032	45.58	43.64	45.67	43.64	-	-

	AE3009	46.59	45.23	46.60	45.24	-	-
	B316	44.29	40.81	44.31	40.83	-	-
	FCS006	46.11	44.39	46.20	44.40	-	-
	FCS014	47.30	46.52	47.34	46.52	-	-
	FD2007	44.18	40.89	44.25	40.91	-	-
	INlla14	44.54	41.15	44.58	41.17	-	-
	MC2021	46.53	45.17	46.50	45.18	-	-
	NC2007	46.16	44.20	46.22	44.21	-	-
	P6B7	44.70	42.54	44.69	42.55	-	-
	P18	43.43	39.55	43.43	39.57	-	-
	Su6	43.85	40.37	43.78	40.39	-	-
	VCB2001	46.15	44.19	46.09	44.20	-	-
	VCB2006	44.79	41.71	44.88	41.72	-	-
	XBB1001	44.51	41.10	44.60	41.12	-	-
	XPD2006	46.02	43.93	45.97	43.94	-	-
Butyrivibrio sp.	AC2005	44.66	40.71	44.94	40.74	-	-
	AD3002	45.58	42.48	45.73	42.51	-	-
	AE3004	44.09	40.21	44.21	40.23	-	-
	AE3006	45.34	42.02	45.56	42.05	-	-
	FC2001	45.45	42.34	45.63	42.37	-	-
	LC3010	43.86	40.19	43.91	40.21	-	-
	MB2005	45.38	41.90	45.52	41.93	-	-
	NC2002	44.23	40.76	44.30	40.77	-	-
	OB235	45.64	42.23	45.75	42.27	-	-
	VCD2006	45.41	41.79	45.59	41.83	-	-
	WCD2001	45.59	42.56	45.75	42.59	-	-
	WCD3002	45.30	42.04	45.42	42.06	-	-
	WCE2006	43.80	40.08	43.93	40.10	-	-
	XPD2002	45.49	42.45	45.73	42.47	-	-
P. ruminis	A12-1	43.11	38.97	43.39	39.06	40.35	37.04

	ACV-9	42.99	39.12	43.35	39.20	40.35	36.75
	AD2017	42.80	38.55	43.17	38.63	-	-
	C4	43.04	39.02	43.35	39.10	40.43	36.73
	CF1b	43.99	39.75	44.30	39.80	-	-
	HUN009	42.99	38.98	43.21	39.05	40.22	36.90
	JW11	43.12	39.04	43.32	39.12	40.42	37.14
	LB2011	43.04	39.13	43.31	39.22	40.43	36.79
	MD2005	42.76	38.43	43.23	38.47	-	-
	OR37	43.06	38.88	43.25	38.95	-	-
P. xylanivorans	Sp 49	43.82	39.14	43.82	39.14	-	-
	ACV2	43.72	38.86	43.72	38.86	-	-
	AR14	43.50	38.98	43.50	38.98	-	-
	Bu21	43.44	38.96	44.05	39.86	-	-
	MZ5	43.69	39.24	43.83	38.91	-	-
	MZ8	44.05	39.86	43.44	38.98	-	-
	NOR37	43.44	38.98	43.44	38.96	-	-
	YE44	43.83	38.91	43.69	39.24	-	-

Supplementary Table 2 - Numbers of inparalogous genes found in each strain, and the total in their respective clade. Inparalogous gene affiliations were found using OrthAgogue with an E-value cutoff of 1×10^{-6}.

Strain/clade	Number of inparalogous genes
***B. fibrisolven*s classical clade total**	**460**
D1	53
AR40	51
MC2013	49
AB2020	46
NC3005	43
WTE3004	40
YRB2005	37
MD2001	36
TB	36
ND3005	35
FE2007	34
***B. hungatei* classical clade total**	**438**
YAB3001	86
M55	46
NK4A153	43
AE2005	42
AE3003	42
LB2008	41
XBD2006	34
INlla21	30
JK615	27
INlla18	27
MB2003	20
***B. proteoclasticus* classical clade total**	**920**
FCS014	178
AE2032	90
INlla14	58
P6B7	57

MC2021	54
P18	49
VCB2001	48
XBB1001	43
AE3009	40
NC2007	40
B316	38
AE2015	37
VCB2006	36
Su6	35
FCS006	32
XPD2006	32
FD2007	31
***Butyrivibrio* sp. classical clade total**	**778**
AC2005	92
AE3004	90
LC3010	65
OB235	62
WCD3002	55
WCE2006	55
VCD2006	54
XPD2002	52
AE3006	51
AD3002	50
MB2005	50
FC2001	47
WCD2001	44
NC2002	33
Butyrivibrio genus total	**2,596**
***P. ruminis* classical clade total**	**305**

OR37	61
CF1b	30
MD2005	30
AD2017	29
JW11	25
A12-1	19
C4	19
LB2011	19
HUN009	19
ACV-9	16
***P. xylanivorans* classical clade total**	**259**
ACV-2	55
49	44
YE44	43
MZ5	41
AR14	38
Bu21	34
NOR37	21
MZ8	21
Pseudobutyrivibrio genus total	**564**

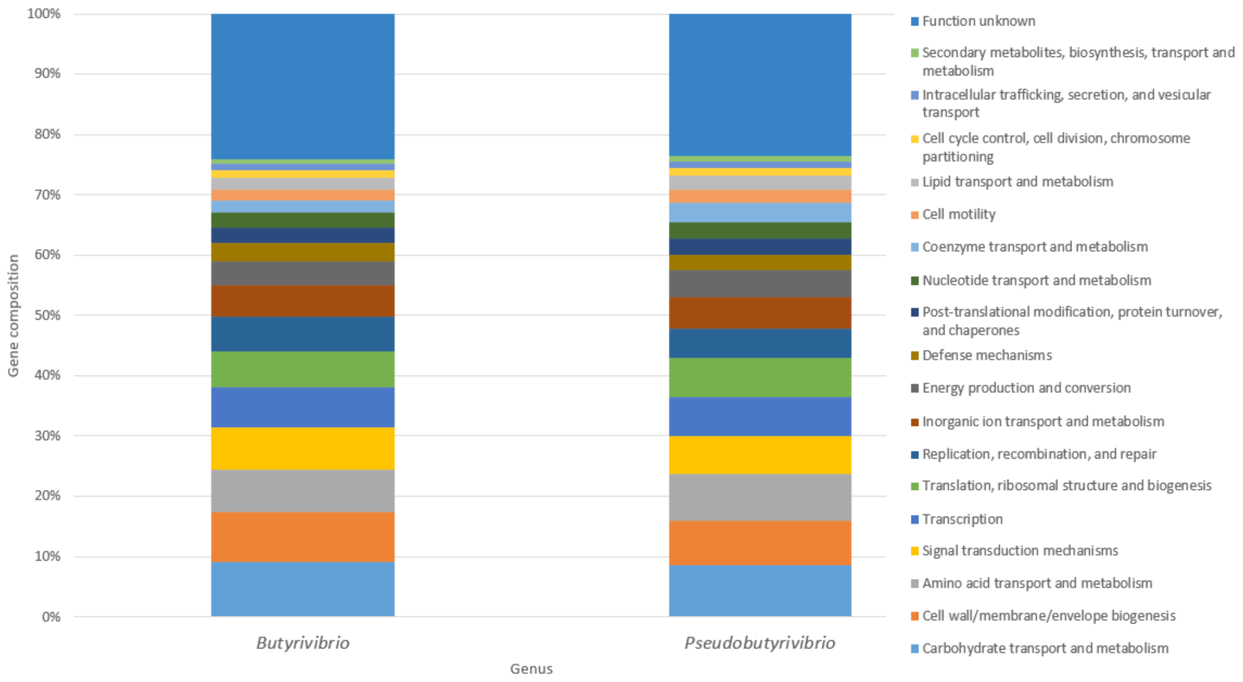

Supplementary Fig. 1 - Functional annotation of *Butyrivibrio* and *Pseudobutyrivibrio* divided into genera. Gene functionality is sorted by colour, as indicated by the key. Annotation was performed by EggNOG (Huerta-Cepas *et al*., 2015, http://eggnogdb.embl.de/#/app/emapper).

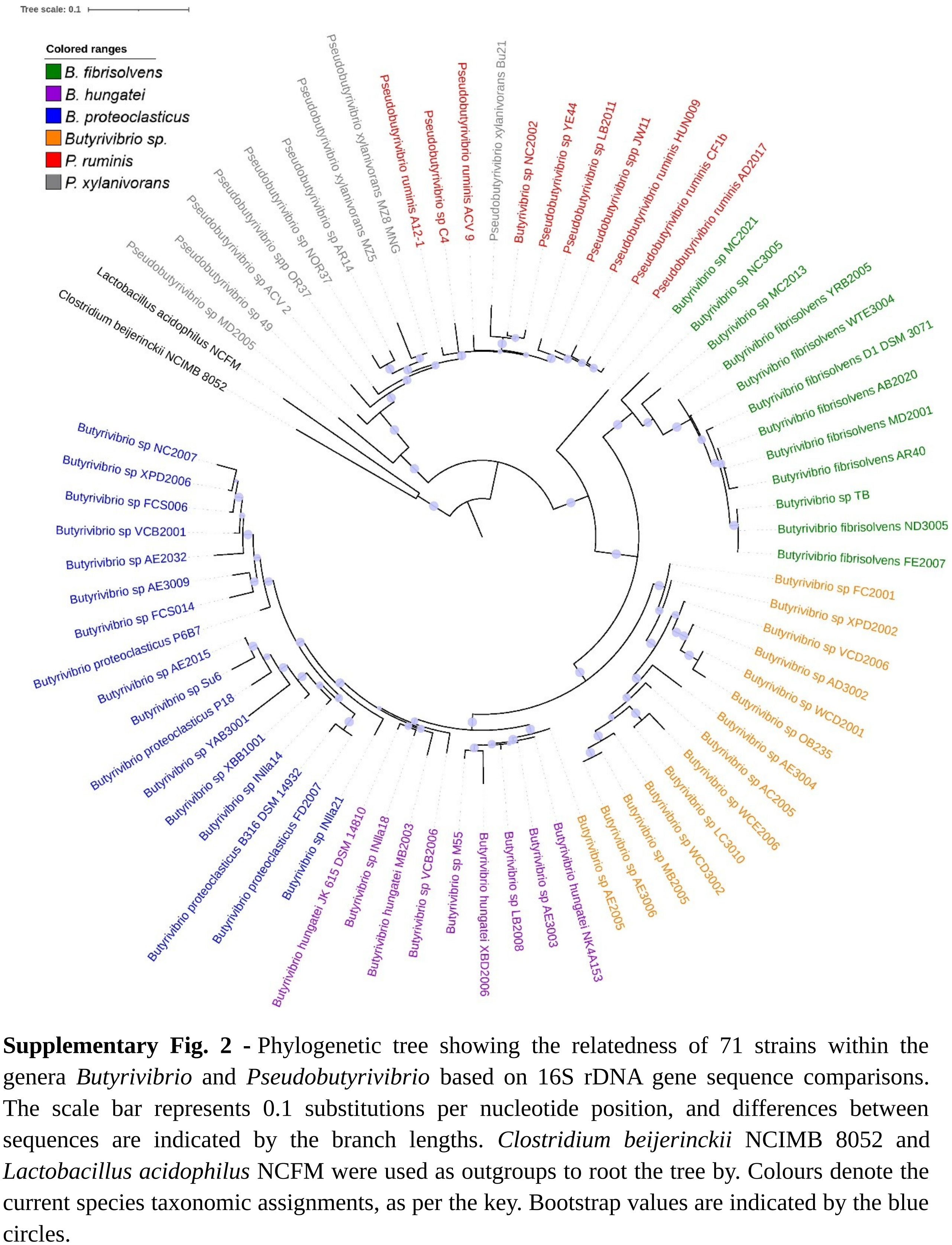

Supplementary Fig. 2 - Phylogenetic tree showing the relatedness of 71 strains within the genera *Butyrivibrio* and *Pseudobutyrivibrio* based on 16S rDNA gene sequence comparisons. The scale bar represents 0.1 substitutions per nucleotide position, and differences between sequences are indicated by the branch lengths. *Clostridium beijerinckii* NCIMB 8052 and *Lactobacillus acidophilus* NCFM were used as outgroups to root the tree by. Colours denote the current species taxonomic assignments, as per the key. Bootstrap values are indicated by the blue circles.

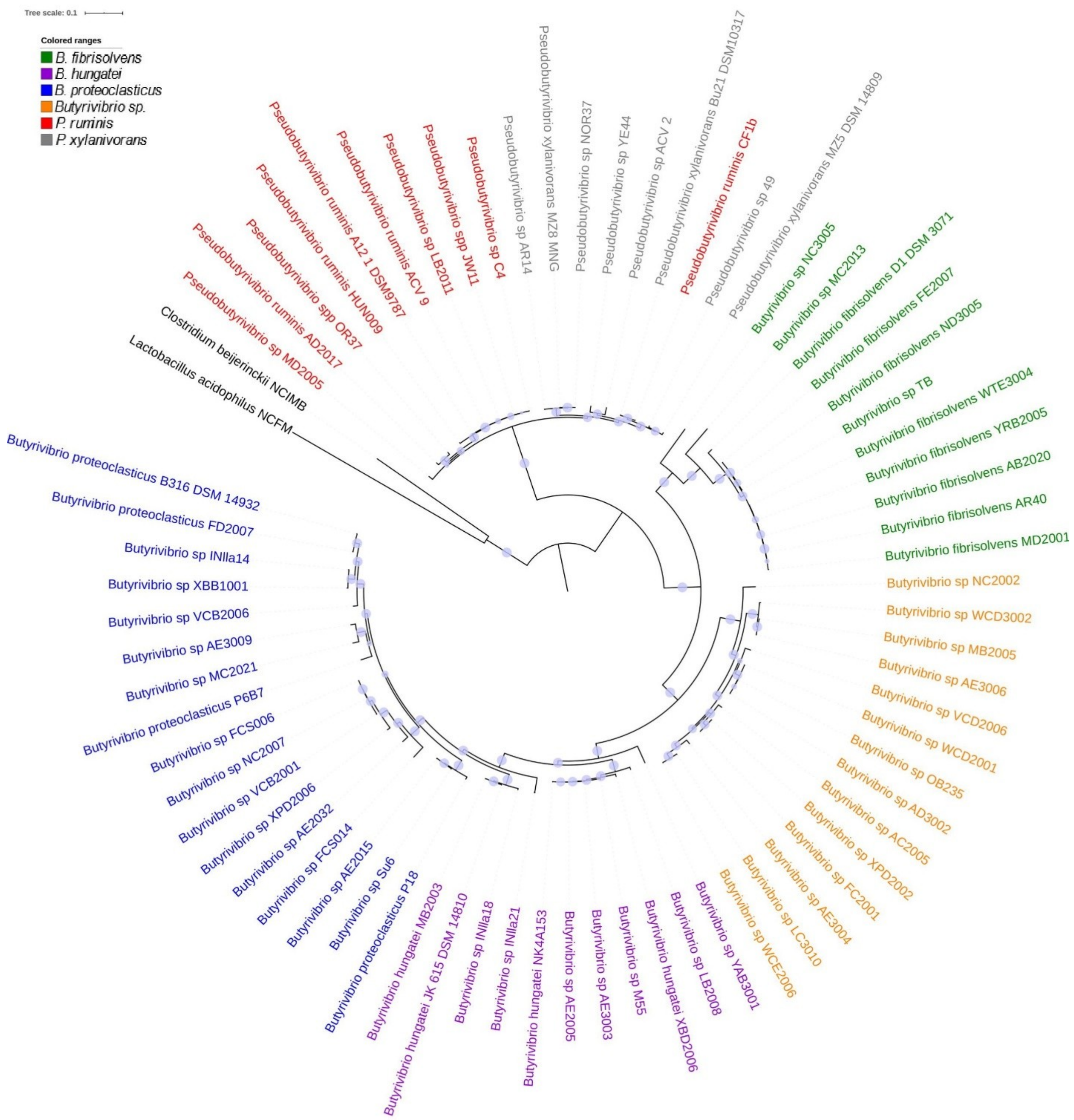

Supplementary Fig. 3 - Phylogenetic tree showing the relatedness of 71 strains within the genera *Butyrivibrio* and *Pseudobutyrivibrio* based on 40 marker gene sequence comparisons. The scale bar represents 0.1 substitutions per nucleotide position, and differences between sequences are indicated by the branch lengths. *Clostridium beijerinckii* NCIMB 8052 and *Lactobacillus acidophilus* NCFM were used as outgroups to root the tree by. Colours denote the current species taxonomic assignments, as per the key. Bootstrap values are indicated by the blue circles.

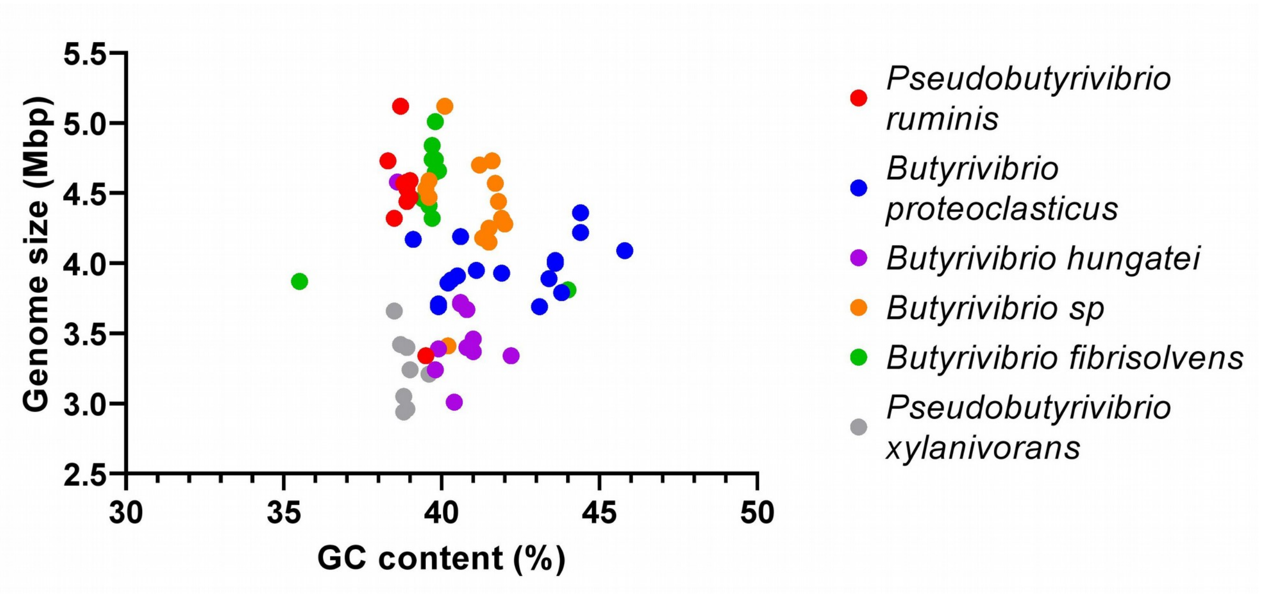

Supplementary Fig. 4 - Scatter plot of GC content (%) vs genome size (Mbp) for 71 strains of *Butyrivibrio* and *Pseudobutyrivibrio*. Colours indicate the groups determined by classical taxonomy and 40 marker phylogeny, as indicated by the key.

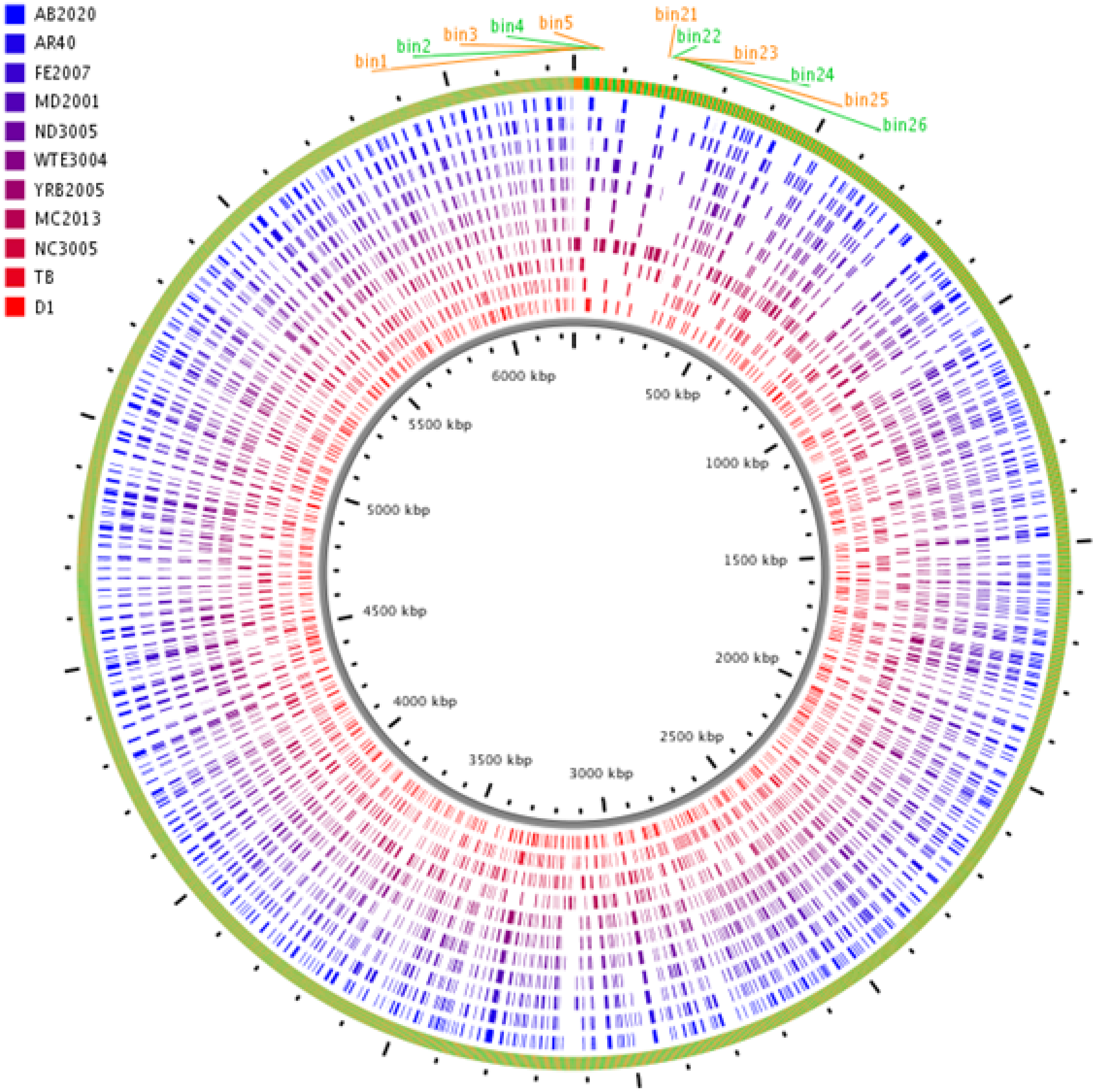

Supplementary Fig. 5 - ClustAGE Plot comparing the distribution of accessory genomic elements (AGEs) for all strains phylogenetically grouped within *Butyrivibrio fibrisolvens* using classical taxonomy. Strains are represented as indicated by the key in each of the tracks. The outer ring (alternating green and orange) indicates individual AGEs, ordered by size as indicated by the inner scale ring. Bin elements can also be observed in this outer ring. The minimum AGE size represented is 1500bp.

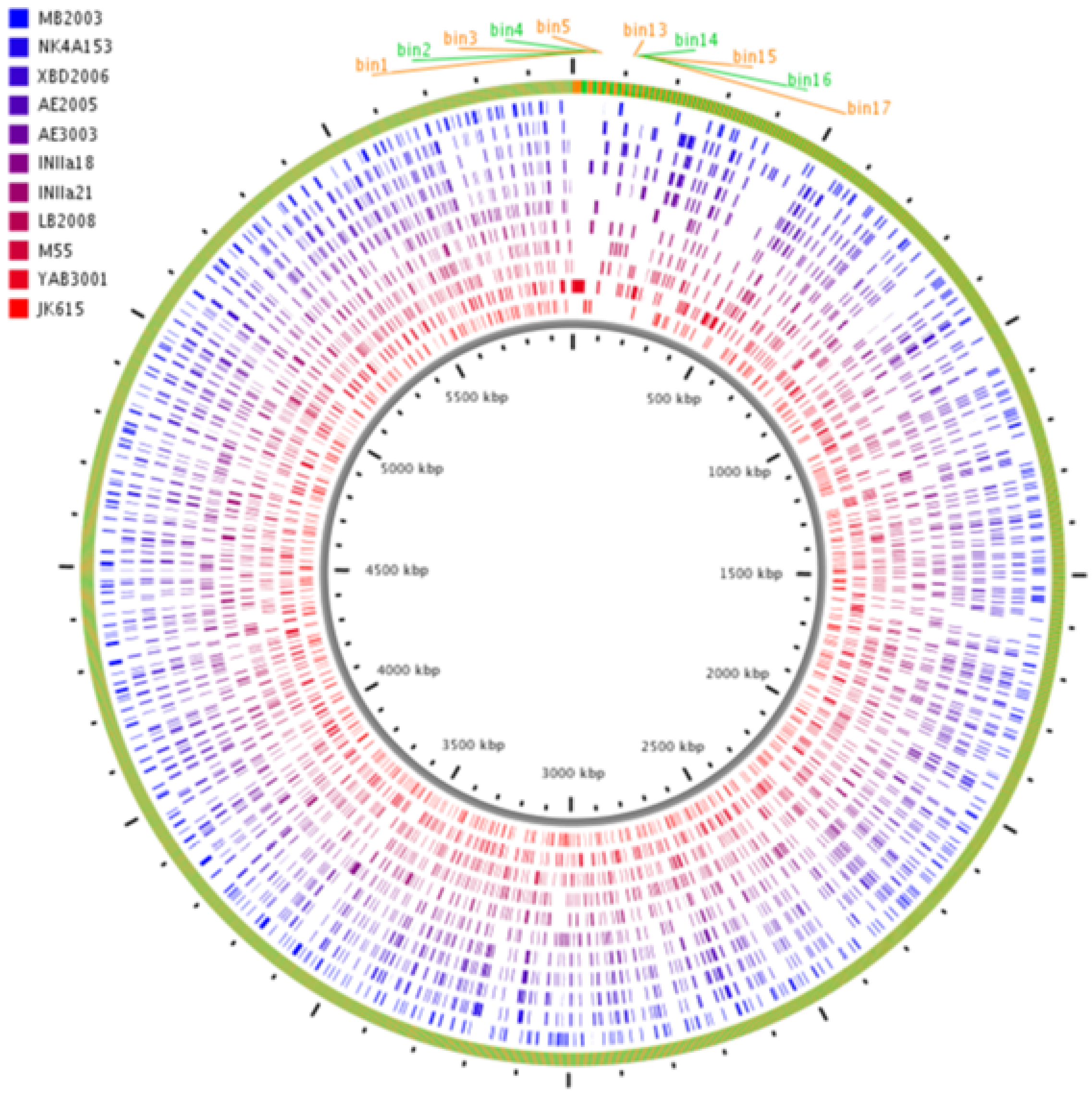

Supplementary Fig. 6 - ClustAGE Plot comparing the distribution of accessory genomic elements (AGEs) for all strains phylogenetically grouped within *Butyrivibrio hungatei* using classical taxonomy. Strains are represented as indicated by the key in each of the tracks. The outer ring (alternating green and orange) indicates individual AGEs, ordered by size as shown by the inner scale ring. Bin elements can also be observed in this outer ring. The minimum AGE size represented is 1500bp.

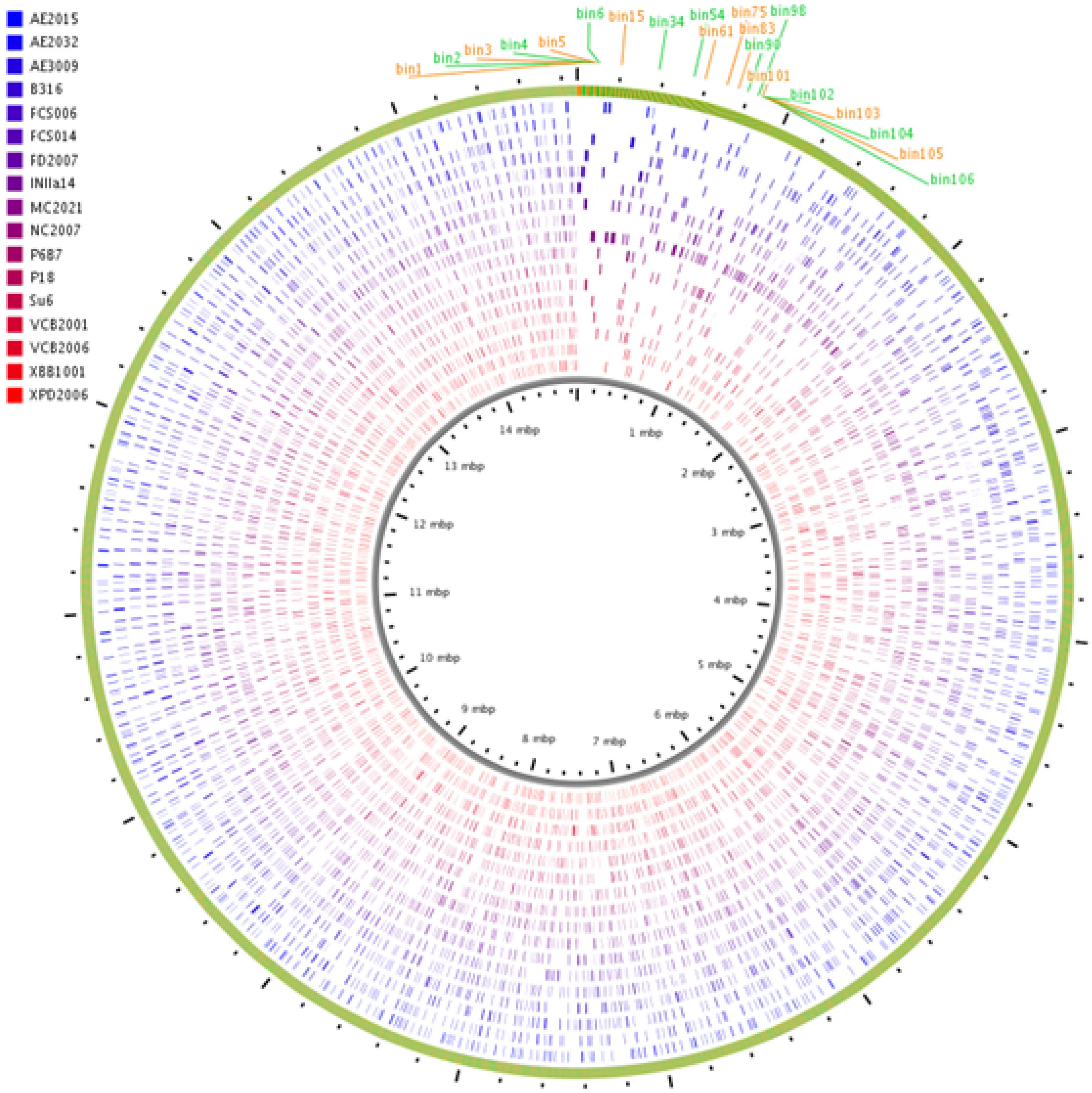

Supplementary Fig. 7 - ClustAGE Plot comparing the distribution of accessory genomic elements (AGEs) for all strains phylogenetically grouped within *Butyrivibrio proteoclasticus* using classical taxonomy. Strains are represented as indicated by the key in each of the tracks. The outer ring (alternating green and orange) indicates individual AGEs, ordered by size as indicated by the inner scale ring. Bin elements can also be observed in this outer ring. The minimum AGE size represented is 1500bp.

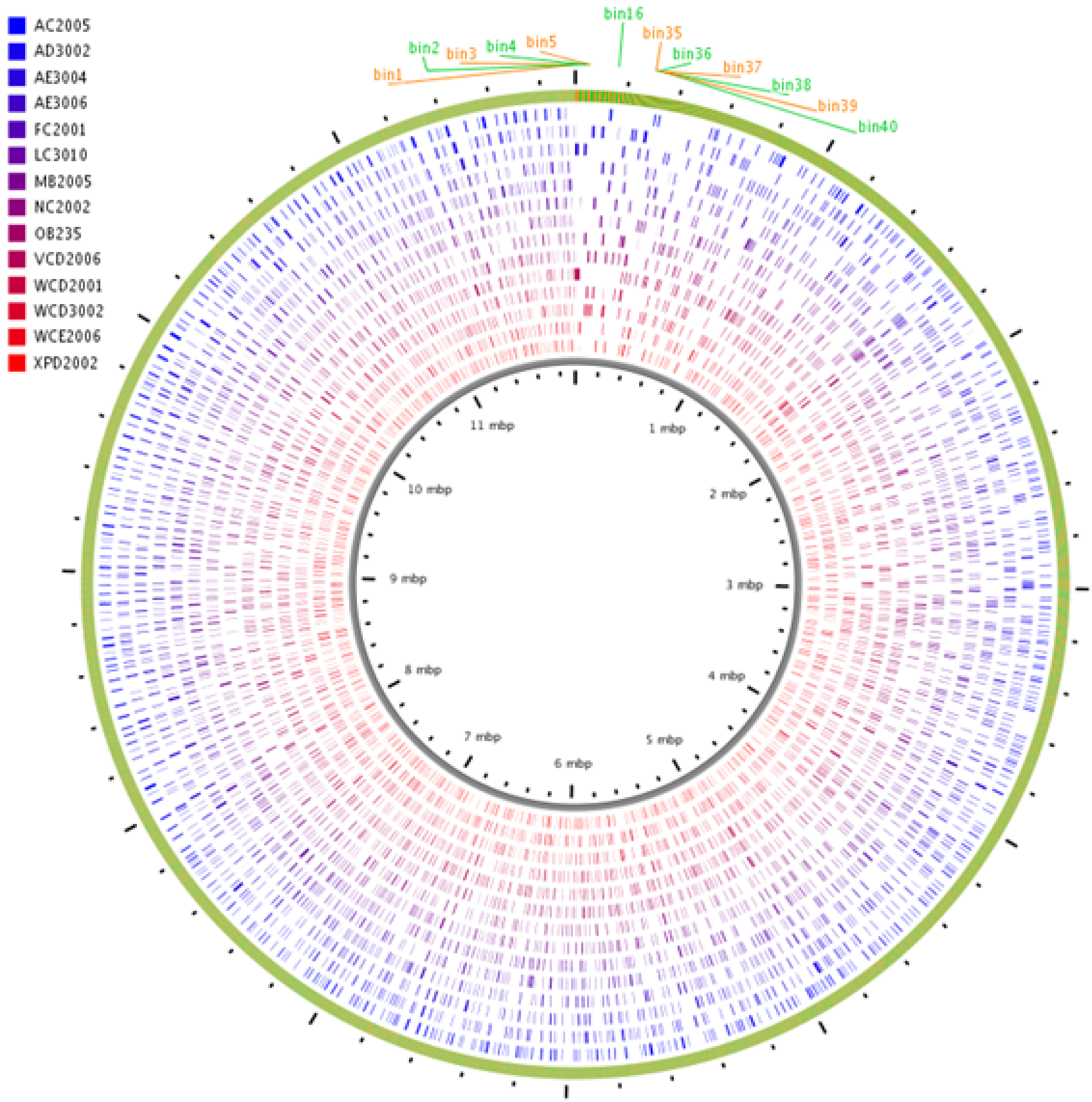

Supplementary Fig. 8 - ClustAGE Plot comparing the distribution of accessory genomic elements (AGEs) for all strains phylogenetically grouped within the *Butyrivibrio sp.* Group using classical taxonomy. Strains are represented as indicated by the key in each of the tracks. The outer ring (alternating green and orange) indicates individual AGEs, ordered by size as indicated by the inner scale ring. Bin elements can also be observed in this outer ring. The minimum AGE size represented is 1500bp.

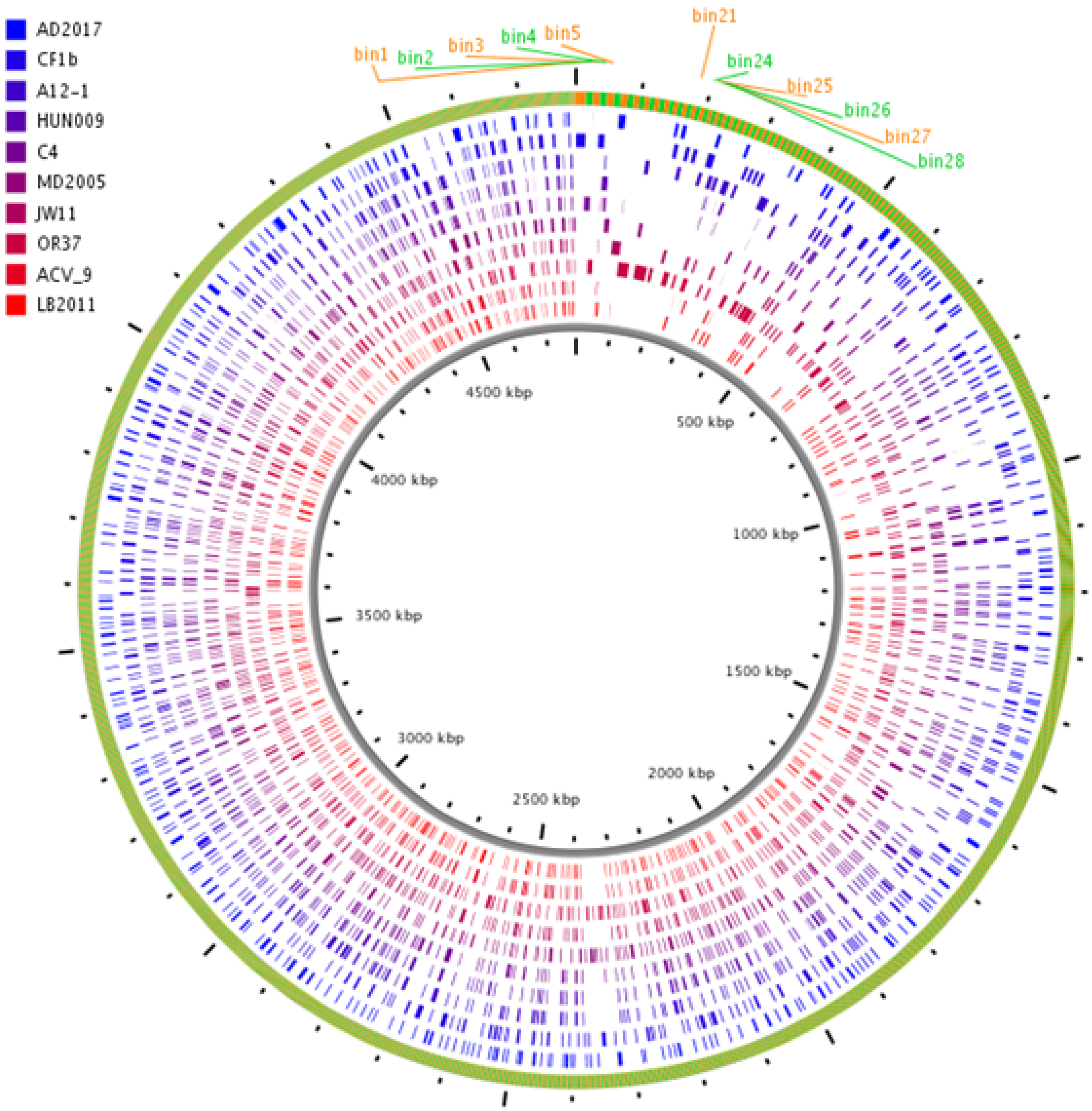

Supplementary Fig. 9 - ClustAGE Plot comparing the distribution of accessory genomic elements (AGEs) for all strains phylogenetically grouped within the *Pseudobutyrivibrio ruminis* using classical taxonomy. Strains are represented as indicated by the key in each of the tracks. The outer ring (alternating green and orange) indicates individual AGEs, ordered by size as indicated by the inner scale ring. Bin elements can also be observed in this outer ring. The minimum AGE size represented is 1500bp.

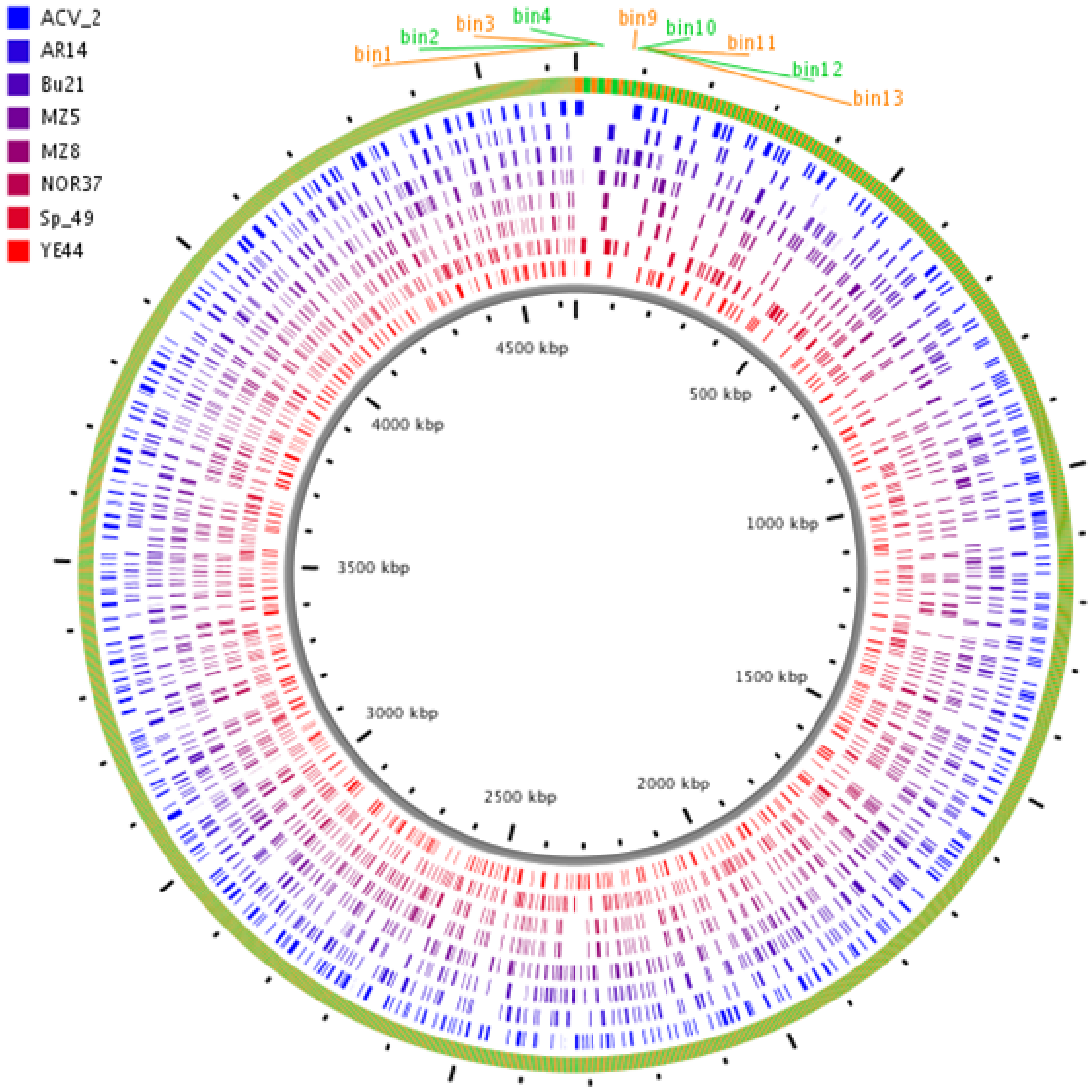

Supplementary Fig. 10 - ClustAGE Plot comparing the distribution of accessory genomic elements (AGEs) for all strains phylogenetically grouped within the *Pseudobutyrivibrio xylanivorans* using classical taxonomy. Strains are represented as indicated by the key in each of the tracks. The outer ring (alternating green and orange) indicates individual AGEs, ordered by size as indicated by the inner scale ring. Bin elements can also be observed in this outer ring. The minimum AGE size represented is 1500bp.

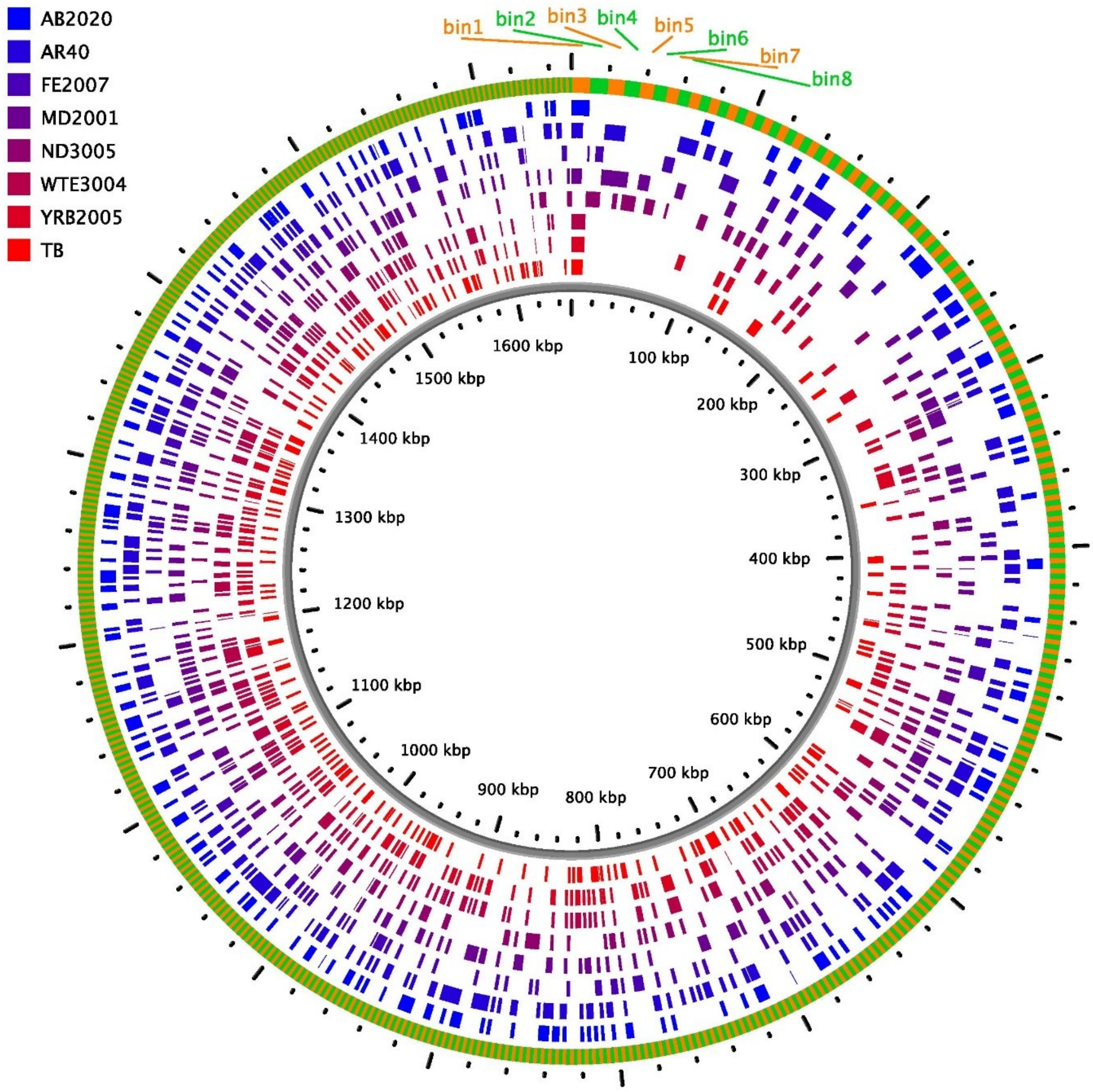

Supplementary Fig. 11 - ClustAGE Plot comparing the distribution of accessory genomic elements (AGEs) for all strains grouped by ANI within the *B. fibrisolvens* group. Strains are represented as indicated by the key in each of the tracks. The outer ring (alternating green and orange) indicates individual AGEs, ordered by size as indicated by the inner scale ring. Bin elements can also be observed in this outer ring. The minimum AGE size represented is 1500bp.

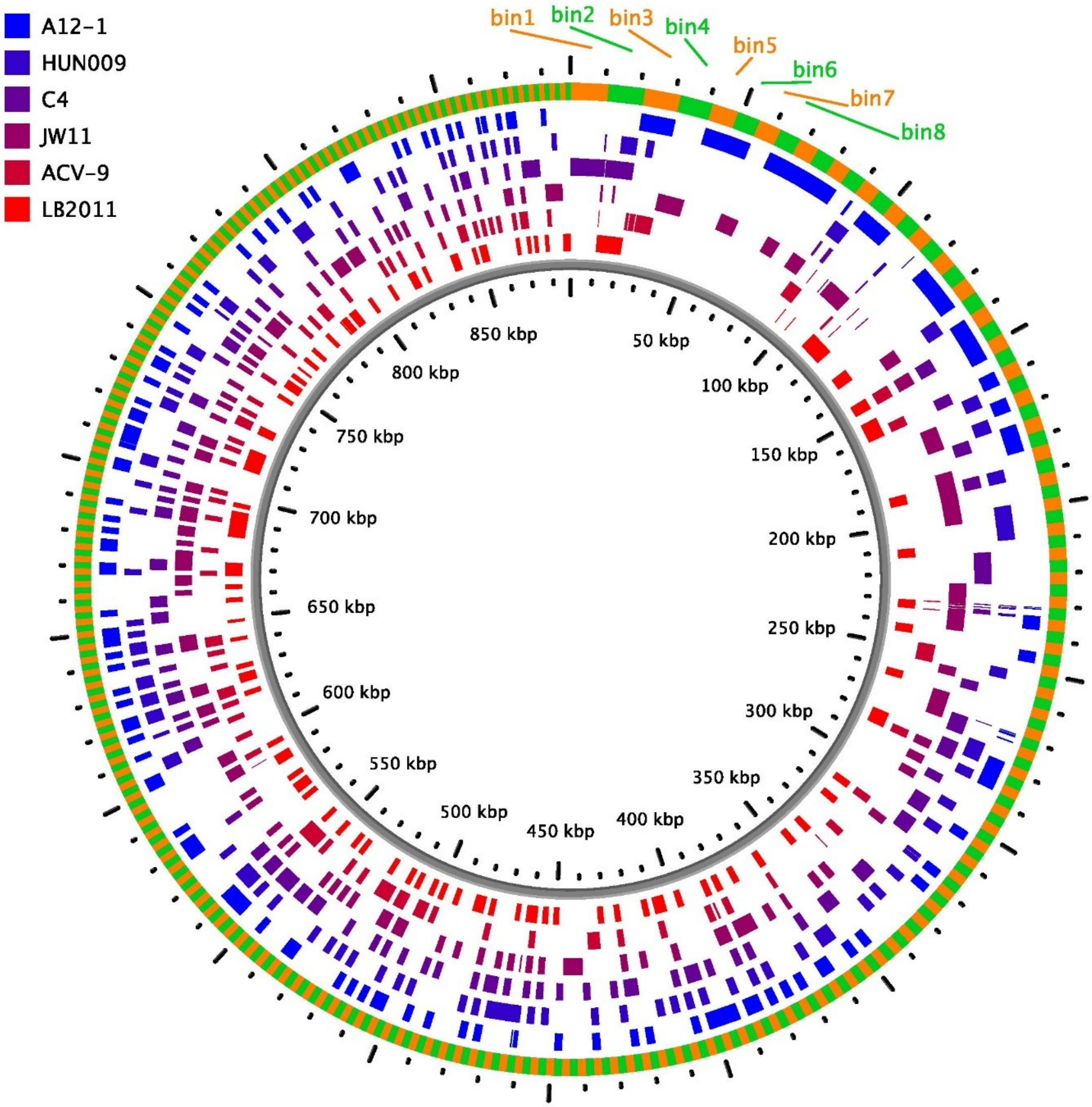

Supplementary Fig. 12 - ClustAGE Plot comparing the distribution of accessory genomic elements (AGEs) for all strains grouped by ANI within the *P. ruminis* group. Strains are represented as indicated by the key in each of the tracks. The outer ring (alternating green and orange) indicates individual AGEs, ordered by size as indicated by the inner scale ring. Bin elements can also be observed in this outer ring. The minimum AGE size represented is 1500bp.

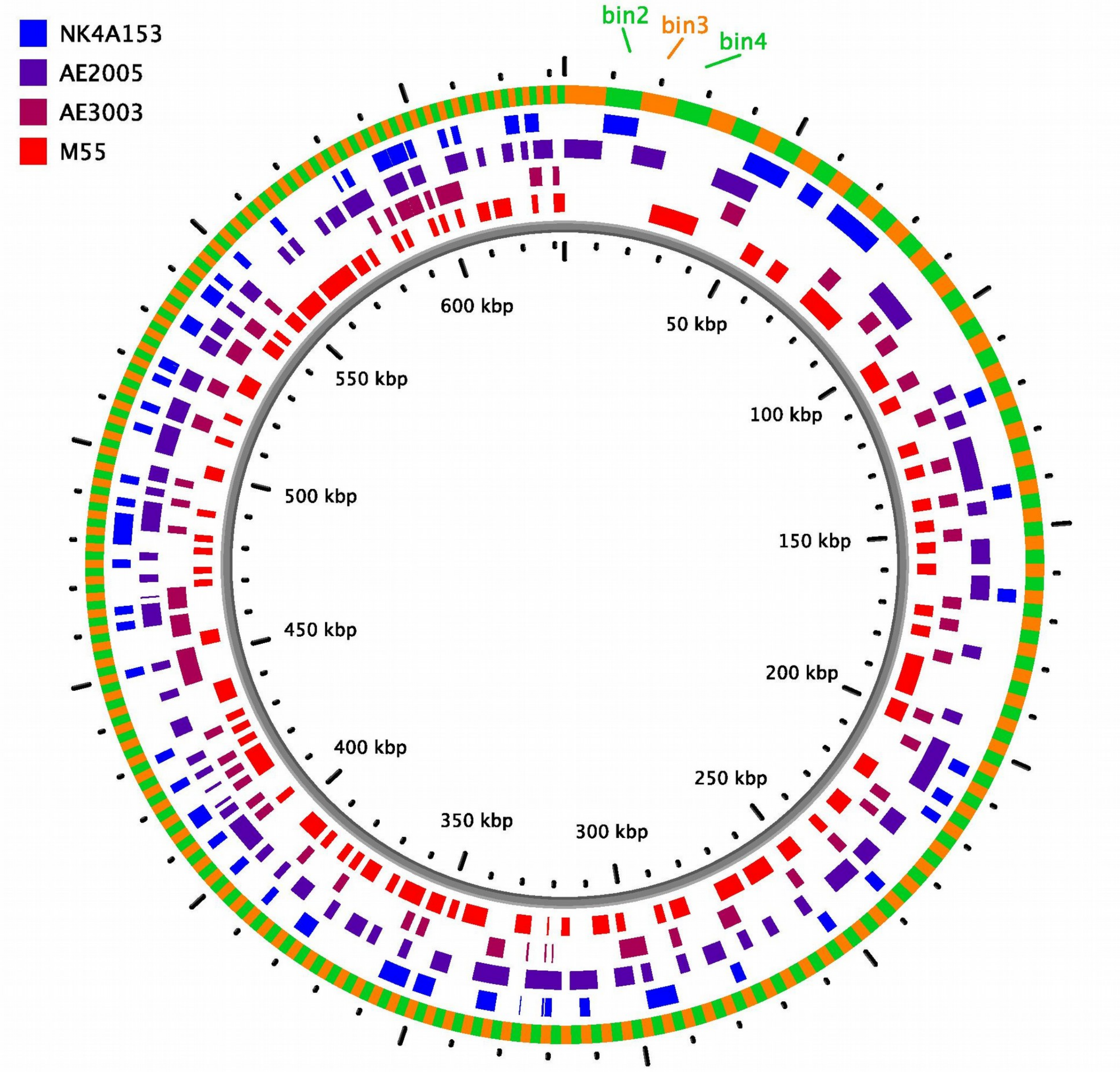

Supplementary Fig. 13 - ClustAGE Plot comparing the distribution of accessory genomic elements (AGEs) for all strains grouped by ANI within the *B. hungatei* group. Strains are represented as indicated by the key in each of the tracks. The outer ring (alternating green and orange) indicates individual AGEs, ordered by size as indicated by the inner scale ring. Bin elements can also be observed in this outer ring. The minimum AGE size represented is 1500bp.

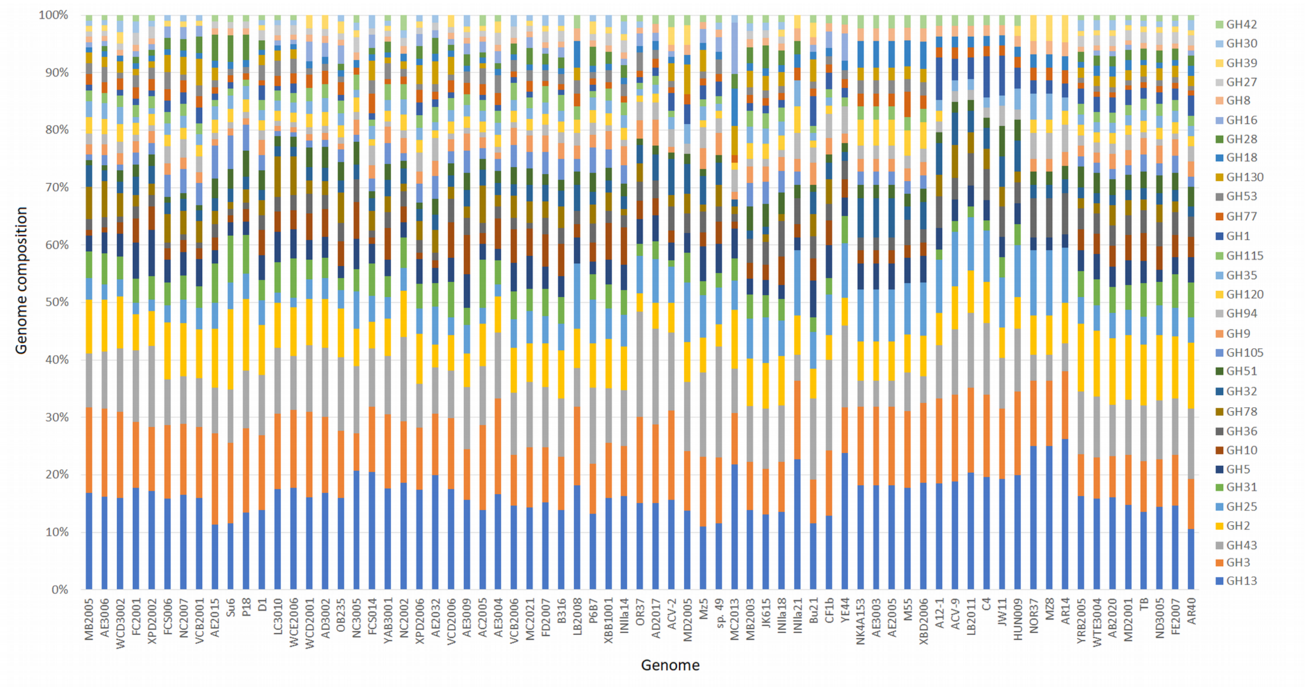

Supplementary Fig. 14 - Proportions of the most abundant glycosyl hydrolase (GH) families found in 71 strains of *Butyrivibrio* and *Pseudobutyrivibrio*. GH families annotated by dbCan metaserver (Yin *et al.*, 2012).
